# Supplementary material for: Innovative assembly strategy contributes to understanding the evolution and conservation genetics of the endangered Solenodon paradoxus from the island of Hispaniola
Source: Gigascience. 2018 Mar 16;7(6):giy025. doi: 10.1093/gigascience/giy025 (PMC6009670; doi:10.1093/gigascience/giy025)

GigaScience

Innovative assembly strategy contributes to understanding the evolution and conservation genetics of the endangered Solenodon paradoxus from the island of Hispaniola

--Manuscript Draft--

**Manuscript Number:**	GIGA-D-17-00182R2
**Full Title:**	Innovative assembly strategy contributes to understanding the evolution and conservation genetics of the endangered Solenodon paradoxus from the island of Hispaniola
**Article Type:**	Research
**Funding Information:**	National Science Foundation (1432092) — Dr. Taras K Oleksyk<br>Ministry of Education and Science of the Russian Federation (Mega-grant 11.G34.31.0068) — Dr. Stephen J O'Brien<br>Saint Petersburg State University (1.50.1623.2013) — Dr. Stephen J O'Brien
**Abstract:**	Solenodons are insectivores living in Hispaniola and Cuba that form an isolated branch in the tree of placental mammals highly divergent from other eulipothyplan insectivores The history, unique biology and adaptations of these enigmatic venomous species could be illuminated by the availability of genome data, but a whole genome assembly for solenodons has not been previously performed, partially due to the difficulty in obtaining samples from the field. Island isolation and reduced numbers have likely resulted in high homozygosity within the Hispaniolan solenodon (Solenodon paradoxus), thus we tested the performance of several assembly strategies on the genome of this genetically impoverished species. The string-graph based assembly strategy seemed a better choice compared to the conventional de Bruijn graph approach, due to the high levels of homozygosity, which is often a hallmark of endemic or endangered species. A consensus reference genome was assembled from sequences of five individuals from the southern subspecies (S. p. woodi). In addition, we obtained additional sequence from one sample of the northern subspecies (S. p. paradoxus). The resulting genome assemblies were compared to each other, and annotated for genes, with a specific emphasis on venom genes, repeats, variable microsatellite loci and other genomic variants. Phylogenetic positioning and selection signatures were inferred based on 4,416 single copy orthologs from 10 other mammals. We estimated that solenodons diverged from other extant mammals 73.6 Mya. Patterns of SNP variation allowed us to infer population demography, which supported a subspecies split within the Hispaniolan solenodon at least 300 Kya.
**Corresponding Author:**	Taras K Oleksyk, Ph.D.<br>UniverSud Paris<br>Mayaguez, Puerto Rico PUERTO RICO
**Corresponding Author Secondary Information:**	
**Corresponding Author's Institution:**	UniverSud Paris
**Corresponding Author's Secondary Institution:**	
**First Author:**	Kirill Grigorev, M.S.
**First Author Secondary Information:**	
**Order of Authors:**	Kirill Grigorev, M.S.<br>Sergey Kliver, M.S.<br>Pavel Dobrynin, Ph.D.<br>Aleksey Komissarov, Ph.D.

	Walter Wolfsberger, M.S.
	Ksenia Krasheninnikova, Ph.D.
	Yashira M Afanador-Hernández, M.S.
	Adam L. Brandt, Ph.D.
	Liz A Paulino, B.S.
	Rosanna Carreras, Ph.D.
	Luis E Rodríguez, Ph.D.
	Adrell Núñez, DVM
	Jessica R. Brandt, Ph.D.
	Filipe Silva, M.S.
	Audrey J Majeske, Ph.D.
	David Hernández-Martich, Ph.D.
	Agostinho Antunes, Ph.D.
	Stephen J O'Brien
	Alfred L Roca, Ph.D.
	Juan Carlos Martinez-Cruzado, Ph.D.
	Taras K Oleksyk, Ph.D.
**Order of Authors Secondary Information:**	
**Response to Reviewers:**	Dear Dr. Edmunds<br><br>Thank you very much for getting back to me so quickly. I carefully went through your comments and addressed all the issues you have raised. Here are the answers to your comments line-by-line. I hope that you will find these satisfactory, but if anything outstanding remains, please let me know as soon as possible.<br><br>Sincerely yours<br><br>Taras Oleksyk, UPR-M<br><br>Line 134<br>Are there also voucher numbers?<br><br>There are no voucher numbers, as the animals were released on location.<br><br>Line 151<br>This is a bit sparse<br><br>Information is added to M&M as requested<br><br>Line 253<br>Make sure this citation remains<br><br>Citations were fixed with the new addition of the GigaScience database<br><br>Line 267<br>Missing citation in the references<br><br>added<br>E. P. Nawrocki and S. R. Eddy, Infernal 1.1: 100-fold faster RNA homology searches, Bioinformatics 29:2933-2935 (2013).<br><br><br>Line 284

Powered by Editorial Manager® and ProduXion Manager® from Aries Systems Corporation

	Can you make sure the scripts are in GigaDB or zenodo?<br><br>I have asked access to the Progressive Cactus scripts. They are are here:<br>https://zenodo.org/record/1189347#.Wp6Jt2aB3sE<br>can this reference be added to GIGADB?<br><br>Line 652<br>Citation missing in references<br><br>Added citation 12<br><br>Line 682<br>Are there reference numbers for these as please include them? If there are voucher numbers and details please include these too<br><br><br>The text was added: "All the required collection and permits had been obtained before any field work was started. The samples have been collected and exported in compliance with Export Permit No: VAPB-00909 (Dominican Republic Environment and Natural Resources Ministry Viceministry of Protected Areas and Biodiversity Department of Biodiversity) and imported in compliance with CITES/ESA Import permit number 14US84465A/9 (University of Illinois Board of Trustees)."<br><br>Line 689<br>What model of HiSeq? 2500? 4000? How did library construction work? Did you use PE reads and what size?<br><br>Added: "Sequences for S. p. woodi were generated by Illumina HiSeq 2000 (Illumina Inc) with 100bp paired-end reads."<br><br>Line 730<br>Make sure this is cited correctly with the full DOI. The curators should give you this when you check the mockup (don't use that). Please also include the accession and zenodo details i f the code is going there<br><br>Added the correct DOI to the database, also the accession numbers<br><br>ENA: NKTL01000000<br>PROJECT: PRJNA368679<br><br>Additional information: http://doi.org/10.5281/zenodo.1185377<br><br>I would also ask to add and addition Zenodo repository here for Progressive Cactus scripts<br>https://zenodo.org/record/1189347#.Wp6Jt2aB3sE
**Additional Information:**	
**Question**	**Response**
Are you submitting this manuscript to a special series or article collection?	No
**Experimental design and statistics**<br><br><br>Full details of the experimental design and statistical methods used should be given in the Methods section, as detailed in our Minimum Standards Reporting Checklist. Information essential to interpreting the data presented should be made available in the figure legends.	Yes

Powered by Editorial Manager® and ProduXion Manager® from Aries Systems Corporation

Have you included all the information requested in your manuscript?	
**Resources**<br><br>A description of all resources used, including antibodies, cell lines, animals and software tools, with enough information to allow them to be uniquely identified, should be included in the Methods section. Authors are strongly encouraged to cite Research Resource Identifiers (RRIDs) for antibodies, model organisms and tools, where possible.<br><br>Have you included the information requested as detailed in our Minimum Standards Reporting Checklist?	Yes
**Availability of data and materials**<br><br>All datasets and code on which the conclusions of the paper rely must be either included in your submission or deposited in publicly available repositories (where available and ethically appropriate), referencing such data using a unique identifier in the references and in the “Availability of Data and Materials” section of your manuscript.<br><br>Have you have met the above requirement as detailed in our Minimum Standards Reporting Checklist?	Yes

Manuscript
Click here to download Manuscript Grigorev et al. GigaScience 2018.main_SCE.TKO.docx

Innovative assembly strategy contributes to understanding the evolution and conservation genetics of the endangered *Solenodon paradoxus* from the island of Hispaniola

Kirill Grigorev [a,1], Sergey Kliver [b,1], Pavel Dobrynin [b], Aleksey Komissarov [b], Walter Wolfsberger [a,c], Ksenia Krasheninnikova [b], Yashira M. Afanador-Hernández [a], Adam L. Brandt [d,e], Liz A. Paulino [f], Rosanna Carreras [f], Luis E. Rodríguez [f], Adrell Núñez [g], Jessica R. Brandt [d,h], Filipe Silva [i,j], J. David Hernández-Martich [k], Audrey J. Majeske [a], Agostinho Antunes [i,j], Alfred L. Roca [d,l], Stephen J. O'Brien [b,m], Juan Carlos Martínez-Cruzado [a] and Taras K. Oleksyk [a,c2]

[a] Department of Biology, University of Puerto Rico at Mayagüez, Mayagüez, Puerto Rico
[b] Theodosius Dobzhansky Center for Genome Bioinformatics, St. Petersburg State University, St. Petersburg, Russia
[c] Biology Department, Uzhhorod National University, Uzhhorod, Ukraine
[d] Department of Animal Sciences, University of Illinois at Urbana-Champaign, Urbana, Illinois, USA
[e] Division of Natural Sciences, St. Norbert College, De Pere, Wisconsin, USA
[f] Instituto Tecnológico de Santo Domingo (INTEC), Santo Domingo, Dominican Republic
[g] Department of Conservation and Science, Parque Zoologico Nacional (ZOODOM), Santo Domingo, Dominican Republic
[h] Department of Biology, Marian University, Fond du Lac, Wisconsin, USA
[i] CIIMAR/CIMAR, Interdisciplinary Centre of Marine and Environmental Research, University of Porto, Terminal de Cruzeiros do Porto de Leixões, Av. General Norton de Matos, s/n, 4450–208 Porto, Portugal
[j] Department of Biology, Faculty of Sciences, University of Porto. Rua do Campo Alegre, 4169-007 Porto, Portugal
[k] Instituto de Investigaciones Botánicas y Zoológicas, Universidad Autónoma de Santo Domingo, Santo Domingo, Dominican Republic
[l] Carl R. Woese Institute for Genomic Biology, University of Illinois at Urbana-Champaign, Urbana, IL, USA
[m] Oceanographic Center, Nova Southeastern University, Fort Lauderdale, Florida, USA

[1] **These authors contributed equally.** ORCIDs: Kirill Grigorev: 0000-0003-3628-0123 Sergey Kliver: 0000-0002-2965-3617.
[2] **Corresponding author:** Taras K. Oleksyk, taras.oleksyk@upr.edu, ORCID: 0000-0002-8148-3918

Abstract

Solenodons are insectivores living in Hispaniola and Cuba that form an isolated branch in the tree of placental mammals highly divergent from other eulipothyplan insectivores The history, unique biology and adaptations of these enigmatic venomous species could be illuminated by the availability of genome data, but a whole genome assembly for solenodons has not been previously performed, partially due to the difficulty in obtaining samples from the field. Island isolation and reduced numbers have likely resulted in high homozygosity within the Hispaniolan solenodon (*Solenodon paradoxus*), thus we tested the performance of several assembly strategies on the genome of this genetically impoverished species. The string-graph based assembly strategy seemed a better choice compared to the conventional de Bruijn graph approach, due to the high levels of homozygosity, which is often a hallmark of endemic or endangered species. A consensus reference genome was assembled from sequences of five individuals from the southern subspecies (*S. p. woodi*). In addition, we obtained additional sequence from one sample of the northern subspecies (*S. p. paradoxus*). The resulting genome assemblies were compared to each other, and annotated for genes, with a specific emphasis on venom genes, repeats, variable microsatellite loci and other

genomic variants. Phylogenetic positioning and selection signatures were inferred based on 4,416 single copy orthologs from 10 other mammals. We estimated that solenodons diverged from other extant mammals 73.6 Mya. Patterns of SNP variation allowed us to infer population demography, which supported a subspecies split within the Hispaniolan solenodon at least 300 Kya.

Keywords

Genome, assembly, de Bruijn, string graph, *Solenodon paradoxus*, Hispaniola, Caribbean, island biogeography, selection drift, isolation, evolution, microsatellites

Background

The only two surviving species of solenodons, found on the two largest Caribbean islands, Hispaniola (*Solenodon paradoxus)* and Cuba (*S. cubanus*), are among the few endemic terrestrial mammals that survived human settlement of these islands. Phenotypically, solenodons somewhat resemble shrews (**Figure 1),** but molecular evidence indicates that they are actually the sister-group to all other extant eulipotyphlan insectivores (hedgehogs, moles, shrews) from which they split in the Cretaceous Period [1–3]. These enigmatic species have various local names in Cuba and Hispaniola, including *oso* (bear), *hormiguero* (ant-eater), *joron* (ferret), *milquí (*or *almiquí)* and *agouta* [4,5], all pointing to the first impression made on the Spanish colonists by its unusual appearance. Today, the Hispaniolan solenodon *(Solenodon paradoxus*) is difficult to find in the wild, both because of its nocturnal activity pattern and the low population numbers. Here, we report the assembly and annotation of the nuclear genome sequences and genomic variation of two subspecies of *S. paradoxus*, using analytical strategies that will allow researchers to formulate hypotheses and develop genetic tools, to assist future studies of evolutionary inference and conservation applications.

S. paradoxus was originally described from a skin and partial skull at the St. Petersburg Academy of Sciences in Russia [6]. It has a large head with a long rostrum with tiny eyes and ears partially hidden by the dusky brown body fur that turns reddish on the sides of the head, throat and upper chest. The tail, legs, snout, and eyelids of the *S. paradoxus* are hairless. The front legs are noticeably more developed, but all four have strong claws useful for burrowing (**Figure 1**). Adult animals measure 49-72 cm in total length, and weigh over 1kg [7]. Solenodons are social animals, they spend their days in extensive underground tunnel networks shared by family groups, and come to the surface at night to hunt small vertebrates and large invertebrates [8]. A unique feature is the *os proboscidis*, a bone extending forward from the nasal opening to support the snout cartilage [9]. Solenodons are venomous mammals that display a fascinating strategy for venom delivery. The second lower incisor of solenodons has a narrow, almost fully enfolded tubular channel, through which saliva secreted by the submaxillary gland flows into the victim [10]. The genus name *Solenodon* means “grooved tooth” in Greek and refers to the shape of this incisor. Although solenodons rarely bite humans, the bites can be very painful (Nicolás Corona, personal communication), and even a small injection of venom has been shown to be fatal to mice in minutes [7]. The chemical composition of solenodon venom has not yet been resolved [11].

Roca et al. 2004 sequenced 13.9 kb of nuclear and mitochondrial sequences of *S. paradoxus*, inferring that solenodon divergence from other eulipotyphlan mammals such as shrews and moles dates back to the Cretaceous Period, ~76 million years ago (Mya), before the mass extinction of the dinosaurs ~66 Mya. Brandt et al. [12] sequenced complete mitogenome sequences of six

Hispaniolan solenodon specimens from southern part of Hispaniola (**Figure 2**), corroborating this conclusion, and estimated that *S. paradoxus* diverged from all other mammals approximately 78 Mya [12]. Other studies have reported similarly deep divergence dates (reviewed in [13]). Whole genome analysis of *S. paradoxus* could provide support and validation to the earlier evolutionary studies.

Morphometric studies suggest that southern and northern Hispaniolan solenodons may be distinctive enough to be considered separate subspecies [2,14,15], a notion supported by recent mitochondrial DNA studies [12,16]. The southern Hispaniolan solenodons had less genetic diversity than those in the north, so that the control region sequences of all five southern specimens (the same individuals used in this study) were identical or nearly identical [12], indicating that Hispaniolan solenodons have a very low level of mitochondrial diversity.

It may now be imperative to study conservation genomics of solenodons, because their extinction would mean the loss of an entire evolutionary lineage whose antiquity goes back to the age of dinosaurs. *S. paradoxus* survived in spectacular island isolation despite the devastating human impact to biodiversity in recent centuries [3,12]. Nevertheless, survival of this species is now threatened by deforestation, increasing human activity, and predation by introduced dogs, cats and mongooses. It is declining in population and its habitat is severely fragmented, and it is listed as endangered by the IUCN Red List of Threatened Species (Red List category B2ab, assessed in 2008; http://www.iucnredlist.org/details/20321/0).

In this study, we assembled the genome of *S. paradoxus* using low coverage genome data (~5x each) from five individuals of *S. paradoxus woodi*. We take advantage of the low individual and population genetic diversity to pool individual data, and apply a string graph assembly approach resulting in a working genome assembly of the *S. paradoxus* genome from the combined paired-end dataset (approximately 26x; **Figure 3**). Our methodology introduces a useful pipeline for genome assembly to compensate for the limited amount of sequencing which, in this instance, performs better than the assembly by a traditional de Bruijn algorithm (SOAPdenovo2) [17]. We employed the string-graph assembler Fermi [18] as a principal tool for contig assembly in conjunction with SSPACE [19] and GapCloser [17] for scaffolding. The resulting genome sequence data was sufficient for high-quality annotation of genes and functional elements, as well as for comparative genomics and population genetic analyses. Prior to this study, the string-graph assembler Fermi [18] has been used only in studies for annotation, or as a complementary tool for *de novo* assemblies made with de Bruijn algorithms [20]. We present and compare genome assemblies for the southern subspecies (*S. p. woodi*) based on several combinations of assembly tools, provide a high-quality annotation of genome features and describe genetic variation in two subspecies (*S. p. woodi* and *S. p. paradoxus),* make inferences about recent evolution and selection

signatures in genes, trace demographic histories, and develop molecular tools for future conservation studies.

Data description

Sample collection and sequencing

Five adult individuals of *S. paradoxus woodi* (NCBI Taxon ID:1906352) from the southern Dominican Republic were collected in the wild following a general field protocol described earlier [12] including two specimens caught from La Cañada del Verraco, and three from the El Manguito location in the Pedernales Province. The captured individuals were visually assessed for obvious signs of disease, weighed, measured, sexed, and released at the capture site, all within 10 minutes of capture. Geographic coordinates were recorded for every location. In addition, one *S. p. paradoxus* (Spa-1) sample was acquired through the collaboration with ZooDom at Santo Domingo, and originated in the Cordillera Septentrional in the northern part of the island. **Figure 2** highlights geographical locations of sample collection points for the samples used in this study.

The five *S. p. woodi* samples were sequenced using Hiseq2000 technology (Illumina Inc.), resulting in an average of 151,783,327 paired-end reads 101 bp long, or 15.33Gb of sequence data, per individual. In addition, DNA extracted from the northern solenodon (*S. p. paradoxus*) Spa-1 was sequenced using MiSeq V3 technology (Illumina Inc.), and produced a total of 52,358,830 paired-end reads, equating to approximately 13.09Gb of sequence data. Only the samples of *S. paradoxus woodi* were used for assembly since the northern subspecies (*S. paradoxus paradoxus*) did not have sufficient coverage for the *de novo* assembly.

Further details about sample collection, DNA extraction, library construction and sequencing can be found in the Methods section. The whole genome shotgun data from this project has been deposited at DDBJ/ENA/GenBank under the accession NKTL00000000. The version described in this paper is version NKTL01000000. The genome data has also been deposited into NCBI under BioProject PRJNA368679, and to the *GigaScience* GigaDB repository [21].

Read correction

After the reduction of adapter contamination with Cookiecutter [22], the k-mer distribution in the reads for the five individuals of *S. paradoxus woodi* was assessed with Jellyfish (Jellyfish, RRID:SCR_005491)[23]. The predicted mean genome coverage was approximately 5x for each sample (**Figure 3**), which is too low for individual de-novo genome assembly. However, because of the extremely low levels of genetic diversity suggested by the earlier study of mitochondrial DNA in the southern subspecies [12], and in order to increase the average depth of coverage, the reads from the five samples were combined into a single data set. As a result, the projected mean genome

coverage for the combined genome assembly was 26x. Error correction was applied with QuorUM (QuorUM, RRID:SCR_011840)[24] using the value k = 31. The k-mer distribution analysis by Jellyfish in the combined and error-corrected data set indicated very low levels of heterozygosity in accordance with the hypothesis (see **Figure 3** legend), allowing use of the combined dataset for the further genome assembly. The genome size has been estimated using KmerGenie [25] to be 2.06Gbp.

Analyses

Assembly tool combinations

We used several alternative combinations of tools to determine the best approach to an assembly of the combined genome data, outlined in **Table 1**. First, the combined libraries of paired-end reads were assembled into contigs with Fermi, a string graph based tool [18]. Second, the same libraries were also assembled with SOAPdenovo2, a de Bruijn graph based tool (SOAPdenovo2 , RRID:SCR_014986)[17]. The optimal k-mer length parameter for SOAPdenovo2 was determined to be k = 35 with the use of KmerGenie [25]. For the scaffolding step we used either SSPACE (SSPACE, RRID:SCR_005056)[19] or the scaffolding module of SOAPdenovo2 [17]. Finally, for all instances, the GapCloser module of SOAPdenovo2 was used to fill in gaps in the scaffolds (GapCloser, RRID:SCR_015026)[17]. After assembly, datasets were trimmed: scaffolds shorter than 1Kbp were removed from the output. In **Table 1**, the four possible combinations of tools used for the assembly are referred to with capital letters A, B, C, and D for brevity. However, SOAPdenovo2 introduces artifacts at the contig construction stage, which it is specifically designed to mitigate at later stages, and SSPACE is not aware of such artifacts [26]. For this reason, the assembly produced by combination D (contig assembly with SOAPdenovo2 and scaffolding with SSPACE) was not reported.

QC and structural comparisons between the assemblies

We used QUAST (QUAST, RRID:SCR_001228)[27] to estimate the common metrics of assembly quality for all combinations of assembly tools: N50 and gappedness (the percentage of *N*s (**Table 1)**). Fermi-assembled contigs (A and B) were overall longer and fewer in number than the SOAPdenovo2 (C and D). The assembly completeness was also evaluated with both BUSCO (BUSCO , RRID:SCR_015008)[28] and CEGMA (CEGMA, RRID:SCR_015055)[29] for completeness of conservative genes. Fermi assemblies (A and B) showed high levels of completeness compared to SOAPdenovo2 (86% vs 42%) at the contig level. However, this difference is partially mitigated at the scaffolding step where SOAPdenovo2 increases completeness for Fermi assembly (A), and more than doubles it for the SOAPdenovo2 assembly (C). To directly

evaluate the quality of all the assemblies we applied REAPR [30]. From the REAPR metrics presented at the bottom part of Table 1, it appears that, even though the scaffolding step has increased the final N50 for the C assembly, it contains significantly more regions with high probability of mis-assemblies (low-scoring regions), less error-free bases, and 3 to 6 times higher number of incorrectly oriented reads compared to the Fermi based assemblies (A and B) (**Table 1**).

We hypothesized that aligning the three genome assemblies to each other will allow us to detect some of these mis-assemblies. A comparison to the best, most closely related genome assembly (i.e., *Sorex araneus*) will reveal several rearrangements that in many cases reflect real evolutionary events. It is reasonable to assume that, if all the rearrangements that are detected are real, and not due to the assembly artifacts, the number of detected rearrangements vs *Sorex* assembly should be the same for all three *Solenodon* assemblies (A, B and C). Following the parsimony principle, an assembly showing rearrangements is also likely to contain the most assembly artifacts. Conversely, we expected that the best of the three assemblies of the *Solenodon* genome should contain the least number of reversals and transpositions when compared to the best available closely related genome (*Sorex araneus*).

To test this hypothesis, the three completed assemblies of *Solenodon* (A, B and C) were aligned to each other, and to the outgroup, which was the *Sorex* genome (SorAra 2.0, NCBI accession number GCA_000181275.2), using Progressive Cactus [31]. Custom scripts were employed to interpret binary output of the pairwise genome by genome comparisons, and the resulting coverage metrics are presented in **Table 2**. In this comparison, all three *Solenodon* genome assemblies had a substantial overlap, and resulted in similar levels of synteny when compared against the *Sorex* reference assembly, but assemblies A and B had the fewest differences with *Sorex*, while assembly C had more differences vs. A, B and *Sorex*. Next, syntenic blocks between each of the three *Solenodon* assemblies (A, B and C) were compared to the *Sorex* assembly, and 50Kbp syntenic blocks were identified using the ragout-maf2 synteny module of the software package *Ragout* [32], and the numbers of scaffolds that contained syntenic block rearrangements were determined. As a result, assembly B had the lowest number of reversals and transpositions when compared to the *S. araneus* reference genome (**Table 2**). Based on the combined results of the evaluations by REAPR [30], Progressive Cactus [31] and Ragout [32], assembly C (generated by the complete SOAPdenovo2 run) was not included in further analysis.

Genome annotation and evaluation of assembly completeness

Repeats in assemblies A and B were identified and soft masked using RepeatMasker (RepeatMasker , RRID:SCR_012954)[33] with the RepBase library [34]. The total percentage of all interspersed repeats masked in the genome was lower than in *S. araneus* (22.53% vs 30.48%). One possible reason could be that a low coverage assembly may perform better in non-repetitive regions.

Alternatively, if the repeat content in *S. paradoxus* is indeed lower, this would have to be evaluated using a higher quality assembly with the use of long read data. The total masked repeat content of the *S. paradoxus* genome including simple/tandem repeats, satellite DNA, low complexity regions, and other elements is presented in **Table 3**. The repeat content can be retrieved from **Database S1**.

The annotation of protein-coding genes was performed using a combined approach that synthesized both homology-based and *de novo* predictions, where *de novo* predictions were used to fill gaps and extend homology-based predictions. Gene annotation was performed for both assemblies (A and B) independently. Proteins of four reference species *S. araneus* (SorAra 2.0, GCA_000181275.2), *Erinaceus europaeus* (EriEur2.0, GCA_000296755.1), *Homo sapiens* (GRCh38.p7) and *Mus musculus* (GRCm38.p4) were aligned to a *S. paradoxus* assembly with Exonerate [35] with a maximum of three "hits" (matches) per protein. The obtained alignments were classified into the top (primary) hit and two secondary hits; the coding sequence (CDS) fragments were cut from each side by 3bp for the top hits and by 9bp for secondary hits. These truncated fragments were clustered and supplied as *hints* (local pieces of information about the gene in the input sequence, such as a likely stretch of coding sequence) of the potentially protein-coding regions to the AUGUSTUS software package (Augustus: Gene Prediction , RRID:SCR_008417)[36], which predicted genes in the soft-masked *Solenodon* assembly. Proteins were extracted from the predicted genes and aligned by HMMER (Hmmer, RRID:SCR_005305)[37] and BLAST (NCBI BLAST , RRID:SCR_004870)[38] to Pfam (Pfam , RRID:SCR_004726)[39] and Swiss-Prot [40] databases, respectively. Genes supported by hits to protein databases and hints were retained; the unsupported sequences were discarded. The annotated genes can be retrieved from **Database S2**.

Assembly B showed a higher support compared to assembly A (91.7% vs 79.2%) for the protein coding gene predictions by extrinsic evidence, even though assembly A had a larger N50 value (**Table 1**). These values were calculated as a median fraction of exons supported by alignments of proteins from reference species to genome (**Figure 4**). In other words, assembly B is more useful for gene predictions, and is likely to contain better gene models that can be used in the downstream analysis. Therefore, based on two lines of evidence: low rearrangement counts (**Table 2**), and high support to gene prediction for the assembly B, it was chosen for the subsequent analyses as the most useful current representation of the *Solenodon* genome.

Non-coding RNA genes

For all non-coding RNA genes except for tRNA and rRNA genes, the search was performed with INFERNAL (Infernal , RRID:SCR_011809) [41] using the Rfam (Rfam , RRID:SCR_007891)[42] BLASTN hits as seeds. The tRNA genes were predicted using tRNAScan-SE (tRNAscan-SE, RRID:SCR_010835)[43], and rRNA genes were predicted with Barrnap ((*BAsic*

Rapid Ribosomal RNA Predictor) version 0.6 (Barrnap, RRID:SCR_015995)[44]). Additionally, RNA genes discovered by RepeatMasker at the earlier stages of the analysis were used to cross-reference the findings of rRNA and tRNA-finding software. The list of the non-coding RNA genes can be accessed in **Database S3**.

Multiple genome alignment, synteny and duplication structure

To compare the *Solenodon* genome assembly with other mammalian genomes, a multiple alignment with genomes of related species was performed using Progressive Cactus [31]. Currently available genomic assemblies of cow (*Bos taurus*, BosTau 3.1.1, NCBI accession number DAAA00000000.2), dog (*Canis familiaris*, CanFam 3.1, GCA_000002285.2), star nosed mole (*Condylura cristata*, ConCri 1.0, GCF_000260355.1), common shrew (*S. araneus*, SorAra 2.0, GCA_000181275.2) and *S. paradoxus woodi* (assembly B from this study) were aligned together, guided by a cladogram representing branching order in a subset of a larger phylogeny (**Figure S1**). We evaluated the *S. paradoxus* coverage by comparing it to the weighted coverages of other genomes in the alignment to the *C. familiaris* genome (**Table 4**). Custom scripts were employed to interpret the binary output of Progressive Cactus ("Cactus") [31]. Cactus genome alignments were used to build a "sparse map" of the homologies between a set of input sequences. Once this sparse map is constructed, in the form of a Cactus graph, the sequences that were initially unaligned in the sparse map are also aligned [31]. Weighted coverage of a genome by genome comparison was calculated by binning an alignment into regions of different coverage and averaging these coverages, with lengths of bins as weights. The weighted coverage of *S. paradoxus* to *C. familiaris* was 1.05, which indicated that the present genome assembly is comparable in quality and duplication structure to other available mammalian assemblies, which are close to each other and are close to 1.0 (**Table 4**).

Detection of single-copy orthologs

Single-copy orthologs (single gene copies) are essential for the evolutionary analysis since they represent a useful conservative homologous set, unlike genes with paralogs, which are difficult to compare across species. The longest polypeptide coded by each gene of *S. paradoxus* and of three other *Eulipotyphla – Erinaceus europaeus, S. araneus, C. cristata* – were aligned to profile hidden Markov models of the TreeFam database (Tree families database , RRID:SCR_013401)[45,46] using HMMER [37]. Top hits from these alignments were extracted and used for assignment of corresponding proteins to families. The same procedure was performed in order to assign proteins to orthologous groups using profile HMMs of orthologous groups of the maNOG subset from the eggNOG database (eggNOG, RRID:SCR_002456)[47] as reference. Orthologous groups and families for which high levels of error rates were observed while testing

assignment of proteins to them were discarded; the rest of the orthologous groups and families were retained for further analysis. Proteins and the corresponding assignments were obtained from the maNOG database for seven other species: *H. sapiens*, *M. musculus*, *B. taurus*, *C. familiaris*, *Equus caballus*, *Mustela putorius furo*, and *Monodelphis domestica*. Inspection of assignments across all the species yielded 4,416 orthologous groups containing single copy orthologous genes (**Database S5**).

Species tree reconstruction and divergence time estimation

We used our genome assembly to infer phylogenetic relationships between *S. paradoxus* and other eutherian species with known genome sequences and estimated their divergence time using the new data. Based on the alignments of the single-copy orthologous proteins for the species included in the analysis, a maximum likelihood tree was built using RAxML [48] with the PROTGAMMAAUTO option and the JTT fitting model tested with 1,000 bootstrap replications. From the codon alignments of single-copy orthologs of the eleven species, 461,539 four-fold degenerate sites were extracted. The divergence time estimation was made by the MCMCtree tool from the software package PAML (PAML, RRID:SCR_014932)[49] with the HKY+G model of nucleotide substitutions and 2,200,000 generations of MCMC (of which the first 200,000 generations were discarded as burn-in). A test for substitution saturation [50,51] was performed using DAMBE6 [52] for both all 3rd codon positions and only 4-fold degenerated sites. In both cases the Iss (index of substitution saturation) was significantly lower than threshold value for both symmetrical and asymmetrical trees indicating low saturation level. Therefore, saturation was not detected for any of the 3d positions nor for the 4-fold degenerated sites.

Divergence times were calibrated using fossil-based priors associated with mammalian evolution, listed in **Table 5** and based on [53–56]. FigTree [57] was used to plot the resulting tree, shown in **Figure 5**. According to this analysis, *S. paradoxus* diverged from other mammals 73.6 Mya (95% confidence interval of 61.4-88.2 Mya). This is in accordance with earlier estimates based on nuclear and mitochondrial sequences (e.g., [3,12]) as reviewed by Springer et al. [58]. This date is also much older than the timeframe of molecular estimates of divergence times between most island taxa and their closest mainland relatives [59]. Our data supports solenodons forming a sister group to other eulipotyphlans, i.e., hedgehogs, shrews and moles [60–63], with a divergence date as old as splits between some pairs of mammalian orders, such as between rodents and primates, or carnivores and artiodactyls (**Figure 5**).

Positively selected genes

To evaluate signatures of selection in the assembled genomes we used a dataset of 4,416 orthologous groups containing single copy orthologous genes of the mammalian species described earlier. Single copy orthologs were used as a conservative set necessary for comparing coding sequences that only arose one time in order to avoid the uncertainties associated with paralogs and lineage specific gene duplications. First, we translated DNA sequences into amino acids, aligned them in MUSCLE (MUSCLE , RRID:SCR_011812)[64], and then translated back into DNA code using the original nucleotide sequences by PAL2NAL [65]. Genic dN/dS ratios were estimated among the 11 mammalian species (including *Solenodon*) used in constructing the phylogeny represented in **Figure 5**.

To estimate dN/dS ratios, we used the *codeml* module from the PAML package [49]. The dN/dS ratios were calculated over the entire length of a protein coding gene. The branch-site model was not included in the current analysis because of the risk of reporting false positives due to sequencing and alignment errors [66], especially on smaller datasets, and additional uncertainties could be introduced from the lack of power under synonymous substitution saturation and high variation in the GC content [67].

All the single copy orthologs were plotted in the dN to dS coordinates and color-coded according to the 96 Gene Ontology generic categories (**Figure 6**). We retrieved values of dN, dS and w (w=dN/dS) for all single copy orthologs and used human annotation categories to assign all the genes with their gene ontologies (GO) using the Python package *goatools* [68] and the GO Slim generic database [68] to assign the genes to the major GO categories.

The dN/dS values for the 12 genes exhibiting positive selection (**Table 6**) are visible above line showing dN=dS. Three of these genes belong to the plasma membrane GO category (GO:0005886), while cytosol (GO:0005829), mitochondrial electron transport chain (GO:0005739), cytoplasm (GO:0005737) and generation of precursor metabolites (GO:0006091) were represented by one gene each. Five of the genes exhibiting positive selection signatures could not be assigned to GO categories. Some of these are also associated with the plasma membranes (*TMEM56*, *SMIM3*), and one gene (*CCRNL4*) encodes a protein highly similar to the nocturnin, a gene identified as a circadian clock regulator in *Xenopus laevis* [69]. The full list of genes, GO annotations, and associated dN/dS values are listed in **Database S6**.

Traditionally, one of the most commonly used signatures of selection is the ratio of non-synonymous (dN) to synonymous (dS) substitutions, dN/dS [70]. The synonymous rate (dS) expresses the rate of unconstrained, neutral evolution, so that when dN/dS<1, the usual interpretation is that negative selection has taken place on non-synonymous substitutions. Otherwise, when dN/dS>1, the interpretation is that the positive selection is likely to have accelerated the rate of fixation of non-synonymous substitutions. It is possible to quantify the proportion of non-synonymous substitutions that are slightly deleterious from the differences in

dN/dS between rare and common alleles [71][72]. In our comparison, a subset of single copy orthologs dN/dS compared to the 10 mammalian species (**Figure 5**) is estimated to be ~0.18 or 18%, on average, compared for ~0.25 is reported for the human–chimp and ~0.13 reported for the mouse-rat comparisons [73]. In other words, it suggests that up to 82% of all amino acid replacements in *S. paradoxus* are removed by purifying selection [73].

Note that purifying selection is the conservative force in molecular evolution, whereas positive selection is the diversifying force that drives molecular adaptation. Overall the list of positively selected genes is relatively short compared to numbers of positively selected genes reported in other studies (e.g. human to chimpanzee comparison yields several hundreds of human-specific genes under selection [74–76]. This observation could be a consequence of the averaging effect of large comparison group that included mammals very distantly related to solenodons.

The dN/dS ratios can also be used as a proxy to illustrate the rate of evolution for proteins. By looking at the trends in fast evolved genes (dN/dS > 0.25) we can make inferences about the factors that shaped the genome of this species during the millions of years of island isolation. To summarize the functional contributions, we used the PANTHER Overrepresentation Test and GO Ontology database based on the *H. sapiens* (**Table S1**) and *M. musculus* (**Table S2**) genes [77]. Interestingly, genes involved in the inflammatory response and located on cell surfaces were among those overrepresented among the rapidly evolving genes in Solenodon genome compared either to the human or mouse databases (**Table S1 and S2**).

Venom gene identification

Since solenodon is one of very few venomous eutherian mammals, of special interest in the solenodon genome were the putative venom genes. While there was no saliva sample in our possession that could be analyzed for the expressed toxin genes, a comparative genome approach could be applied as an indirect way to find venom genes orthologous to genes expressed in venom for other species. First, we identified 6,534 toxin and venom protein representatives (Tox-Prot) from Uniprot (UniProt , RRID:SCR_002380)[78], and queried them with BLAST against the current *S. paradoxus* genome assembly. The hit scaffolds were then extracted from the AUGUSTUS CDS prediction file. The same Tox-Prot sequences were used for Exonerate with the protein-to-genome model. The hits were used as queries against the NCBI database to ensure gene identity, further examined through phylogenetic analyses with select model mammalian and venom reptile genes (also adding randomly selected sequences for each gene, to reduce clade bias). The retrieved sequences were aligned with MUSCLE [64], followed by a maximum likelihood (WAG+I+G) phylogenetic reconstruction. Hits were matched against their respective references in an alignment and visually inspected.

As a result, we identified 44 gene hits of the 16 most relevant protein venom classes (all present in snakes) in the *S. paradoxus* genome (**Table 7**). Inspection of pairwise MUSCLE alignments of the putative Solenodon venom genes (**Database S7**) with their animal homologs revealed several interesting cues. The putative venom genes could not be confirmed through genomic information alone, yet they cannot be discarded given that they were matched to high homology regions of closely related genes, such as those originally recruited into venom. There were also unusual insertions not found in other species' venom genes. Specifically, an insertion in a serine protease, a gene with a role in coagulation (namely coagulation factor X), is not present in known homologs. The insertion seems to be located at the start of the second exon. This particular gene was further analyzed to understand the insertion and its potential functional consequences (**Figure 7**). Finally, none of the known venom genes from the closest related venomous insectivore (*Blarina brevicauda*) have been found by this study. Our results indicate that a more detailed study of *Solenodon* venom genes using a transcriptome obtained from a fresh saliva sample is needed to address their molecular evolution and function.

Genomic variation and demographic history inference

Once the reference alignment was assembled as a consensus between the sequences obtained from the five *S. p. woodi* individuals, polymorphisms were identified in the six individual genomes by aligning them to the combined reference. Single-nucleotide and short variants and indels were identified in five southern and one northern individual using Bowtie2 (Bowtie , RRID:SCR_005476)[79], SAMtools and Bcftools (SAMTOOLS , RRID:SCR_002105)[80], and VCFtools (VCFtools, RRID:SCR_001235)[81]. The *S. p. woodi* individuals differed from the reference by an average of 1.25 million polymorphisms, and the *S. p. paradoxus* individual differed by 2.65 million from the reference assembly.

Whole solenodon genome SNV rates, defined as a ratio of all observed SNVs to all possible SNV sites in the genome, were calculated and found to be comparatively low relative to other mammals (**Figure 8**) [82–85]. To enable this comparison, the same calculations were employed, where SNVs were not filtered by repetitive regions or mappability mask and the number of possible SNV sites was defined as the genome assembly size minus number of unknown base pairs ('N').

Based on the variation data from the genomes of two subspecies (*S. p. woodi* and *S. p .paradoxus),* we estimated population dynamics using Pairwise Sequentially Markovian Coalescent (PSMC) model [86]. PSMC uses a coalescent approach to estimate changes in population size that allowed us to create a TMRCA distribution across the genome and estimate the effective population size (*Ne*) in recent evolutionary history (e.g., from 10,000 to 1 million years).

Demographic history was inferred separately for *S. p. woodi and S. p. paradoxus,* and the resulting plots revealed differences in demographic histories of the two subspecies (**Figure 9**). Each

southern individual was considered separately and their demographic histories overlapped. The difference in demographic history provides another argument in favor of a subspecies split, as evidenced by distinctly different effective population sizes at least since 300 Kya. According to this analysis, the northern solenodon subspecies currently has a much larger *Ne*, which has expanded relatively recently, between 10,000 – 11,000 years ago (**Figure 9**). Prior to that, it was the southern subspecies (*S. p. woodi*) that had a larger *Ne*. At the same time, the demographic history inferred for both populations showed similar cyclical patterns of expansion and contraction around the mean of 6,000 "effective" individuals for the southern subspecies (*S. p. woodi*) and 3,000 for the northern subspecies (*S. p. paradoxus*). One unusual result of this analysis is that the northern subspecies shows a much lower Ne for all but the most recent time period.

Development of tools to study population and conservation genetics of S. paradoxus

The presence of genome wide sequences of multiple individuals from two subspecies created a possibility for the development of practical tools for conservation genetics of this endangered species. Generally, microsatellite loci are both abundant and widely distributed throughout the genome, while usable loci are characterized by a unique flanking DNA sequence so that a single locus can be independently amplified in many individuals [87–89]. The major advantages of microsatellite markers are well known: codominant transmission, high levels of polymorphisms leading to the high information content, high mutation rates that allow differentiation between individuals or populations within a species, and ease of genotyping. While a genome obtained from one individual can be searched for potentially variable microsatellite loci, this would (1) miss the majority of variable loci not represented in the individual's two chromosomes, and (2) result in many positives that may be monomorphic following laboratory tests (usually by electrophoresis of the amplified fragments from population samples). The availability of several genomes can allow generation of a more comprehensive set of variable markers, while reducing false positives

All three assemblies from this study (A, B and C) were independently analyzed using a short tandem repeat (STR) detection pipeline. A, B and C assemblies were analyzed separately with TRF (Tandem Repeats Finder) to locate and display tandem repeats [90]. Each of the six individual samples from the two solenodon subspecies (five from *S. p. woodi* and one from *S. p. paradoxus*) were aligned to the reference assemblies A, B, and C by Burrow-Wheelers Aligner (Li and Durbin, 2009). Each set of individual alignments was analyzed with HipSTR [91]. Only loci that shared more than 20 reads in the sample alignments were considered for further steps in the search for variable microsatellite loci. The result of this search was saved in a Variant Call Format (VCF) file that includes annotations of all loci that had variation between samples and passed the minimum qualification of the reads parameter: to be successfully identified *in silico* in the data from at least

one individual. The loci that did not pass these criteria were labeled as *unsuccessfully verified* and excluded from the list.

The remaining loci were subjected to additional filtering: all genotypes that had less than 90% posterior probability according to HipSTR [91], genotypes with a flank indel in more than 15% of reads, and genotypes with more than 15% of reads with detected PCR stutter artifacts were discarded. The final set contains loci that have at least two allele calls in two different individuals after filtering have been deposited in the polymorphic microsatellite database (**Database S8**). This database contains a list of variable microsatellites discovered, a total of 1,200 bp flanking sequence for primer construction, and the information on whether and where it was found variable - between subspecies, or within one of the subspecies. We also report the type (di- tri-, etc.), number of repeats, number of variants, % variable, and provide up to 600bp flanking sequence on each side that can be used to develop primer sequences (**Database S8**).

Discussion

In this study, we sequenced and assembled the genome of an endangered Antillean mammal that survived tens of millions of years of island isolation, but nevertheless is currently threatened with extinction due to anthropogenic activities. Our approach demonstrated sequencing, assembly and annotation of a genome of a highly divergent lineage within the placental mammal tree, delivering an important phylogenetically diverse mammalian genome for analysis in a comparative context [92]. Although the full description of genome diversity of this rare enigmatic mammal needs to be further improved with more samples and analyses, our initial assembly of the solenodon genome contributes information and tools for future studies of evolution and conservation. Future studies can combine the current genome annotations with the inclusion of additional genetic and ecological data from further sampling.

With the new genome-wide assembly, we inferred a phylogeny that validates previous estimates of the time of divergence of *Solenodon* from other eulipotyphlan insectivores [3,12], also providing a window into genetic underpinnings of adaptive features, including genes responsible for inflammation and venom, and how these may reflect its adaptation. In addition, we developed tools that will help guide future genome studies as well as conservation surveys of the remaining solenodon populations on the island of Hispaniola. In this study, we have made the first step into the whole-genome analysis of the *Solenodon*. A more complete genome sequence may provide a better picture of its evolutionary history, possible signatures of selection, clues about the genetic basis of adaptive phenotypic features facilitating life on Caribbean islands, and contribute to a better insight into island evolution and possible responses to current and future climate change.

The string graph assembly approach for homozygous genomes

The advantages of the string graph assemblies in our particular case can be understood by looking at the nature of the underlying algorithms. The de Bruijn graph is a mathematical concept that simplifies genome assembly by reducing information from short next generation sequencing reads, of which there can be billions, to an optimized computational problem that can be solved efficiently [93]. However, some information may indeed be lost, as the set of reads is effectively replaced with a set of much shorter k-mers to produce an optimal assembly path. Usually, this is compensated by overwhelming amounts of data in high coverage assemblies, and the difference in effectiveness between this and other types of algorithms, barring speed, becomes less evident. While sequencing becomes cheaper, genome projects continue to rely on the increased high quality coverage, increasing the cost of the sequence data rather than trying to increase the efficacy of the assembly itself. In contrast, the string graph-based algorithms for genome assembly are intrinsically less erroneous than de Bruijn graph based ones, since building and resolving a string graph does not require breaking reads into k-mers and therefore does not sacrifice long-range information [18]. This also helps reduce the probability of mis-assemblies: in theory, any path in a string graph represents a valid assembly [94,95]. String graph based approaches have already been applied successfully to assemblies from high coverage read sets; and one example is the Assemblathon 2 [96]. In projects with lower genome coverage like ours, adoption of a string graph based approach might be of benefit to the genome assembly because it uses more information from the sequences. However, there are two major downsides for its widespread use: (1) it is more computationally intensive than methods utilizing de Bruijn graph algorithms, and (2) the implementation of the string graph model is sensitive to sequence variation, and the effectiveness of this approach may depend on the level of heterozygosity in a DNA sample. It is worth noting that Fermi [18] was primarily intended for variant annotation via *de novo* local assembly, and not for whole genome assembly. Nevertheless, the new genome-wide data produced by our pipeline was sufficient for the comparative analysis, and has been annotated for the genes and repetitive elements, and interrogated for phylogeny, demographic history and signatures of selection. In addition, using the current genome assembly we were able to annotate large transpositions and translocations in the *Solenodon* in relation to the closest available high-quality genome assembly (*S. araneus*).

Potential implications

Comparative genomics

We have taken advantage of the fact that the genome of this mammal shows reduced heterozygosity [12], which made it feasible to combine samples of multiple individuals in order to

provide higher coverage and achieve a better assembly using Illumina reads. The current assembly was performed without the use of mate pair libraries and without high quality DNA, nevertheless it is comparable in quality to other available mammalian assemblies. In terms of contig N50 as a measure of contiguity, our assembly resulted in contig N50 of 54,944 while the most closely related available genome sequences of *Sorex araneus* (SorAra2.0) assembly features a contig N50 of 22,623, and the *Condylura cristata* (ConCri1.0) assembly has contig N50 of 46,163. It should be noted that scaffold N50 values are not to be compared as this study used only paired-end reads, as opposed to *S. araneus* and *C. cristata*. More importantly, the assembly provided complete or partial annotation for more than 95% of the genes based on the evolutionarily-informed expectations of gene content from near-universal single-copy orthologs selected from OrthoDB v9 by BUSCO [28]. Among these, 4416 single copy genes that have clear one-to-one orthologs across species (single copy orthologs) [97,98] were chosen for a subsequent comparative analysis involving genes in different mammalian species.

Specifically, the repetitive composition of the solenodon genome was evaluated. Compared to the estimates based on the reference human genome [99], very conspicuous is the lower numbers of SINEs (no *Alu* elements), and a substantially lower number of LINEs as well. Transpositions and translocations between the genomes of *S. paradoxus* and *S. araneus* were identified; very few rearrangements and translocations between the assembly and the *S. araneus* genome were found. At the same time a higher coverage would be needed to do more detailed analyses, for instance to address the relative length and similarity of indels and copy number polymorphisms between solenodon populations [100].

Evolutionary genomics

Using the nuclear genomes, we were able to confirm earlier divergence time estimates based on sets of genes [3], as well as full mitochondrial sequences [12]. The whole genome analysis points to a split between *Solenodon* and other eulypotiphlans that occurred around 74 Mya (**Figure 5**), which is very close to our earlier estimate of 78 Mya based on the full mitochondrial genome [12]. Our result does not support the 60 Mya point estimate made by a phylogenetic analysis based on sequences of five slowly evolving nuclear genes [13].

Our assembly provided enough gene sequences to gain insights into the evolution of functional elements in the solenodon genome. It is reasonable to suggest that this species historically had low effective population sizes, if they remained close to those estimated by this study: or about 4,000 on average (**Figure 9**). Among the 4,416 single copy orthologs analyzed for dN/dS ratios over the entire length of a protein-coding gene between *S. paradoxus* and 10 other mammals, 12 genes were identified as positively selected. Among these, the majority were membrane proteins, with one gene (*CCRNL4*) similar to a circadian clock regulator (**Table 6**). It is

possible that the short list of the positively selected genes could be a consequence of the large comparison group that included mammals very distantly related to solenodon, and its genes need to be compared with more closely related species, for example once the genome of *S. cubanus* is reported, and better gene annotations for *Sorex araneus* become available.

Solenodon is one of few mammals that use venomous saliva to disable prey. It delivers its venom similarly to snakes — using its teeth to inject venomous saliva into its target. Different approaches could be used to characterize venom genes, such as the use of non-curated databases to widen the search spectrum, which may include different molecules that could be found in *Solenodon*. For example, 6,534 toxin and venom protein representatives can be found in the UniProt database. It is also important to note that the database of venom gene sequences may not include those relevant to solenodons given their deep divergence from any other venomous mammalian species. The venom of *Solenodon* may contain novel protein modifications with unknown potential or application, making it valuable for future detailed characterization.

Genes associated with venom, such as serine proteases involved in coagulation (namely the coagulation factor X) are of major interest, since factor X in solenodon exhibited unusual insertions when compared to its homologs (**Figure 7**). The detection of an unusual insertion in a serine protease has been previously found in another venomous mammalian species, the shrew *Blarina brevicauda*, but in a different gene than in solenodon. The coagulation factor X is involved in the circulatory system and is responsible for activating thrombin and inducing clotting. The insertion in the coagulation factor X gene seems to be a hydrophilic alpha helix with three potential protein-protein interaction sites. It occurs at the end of the region annotated as the signal peptide, while having a signal peptide cleavage site itself at the beginning of its sequence. The factor X protein structure was successfully modeled by Swiss-Model based on the venomous elapid snake *Pseudonaja textilis* (pdb: 4bxs), to have a heavy chain that contains the serine protease activity, which was modeled with a high degree of confidence (**Figure 10**). The venom prothrombin activator has an advantage as a toxin in part due to modifications in inhibition sites, making it difficult to stop its activity. Another advantage is that the molecules are always found in an active form (Kinin). We hypothesize that the insertion could allow a more successful interaction with molecules capable of activating the F10 protein. In mice, venom extracted from solenodons and venom prothombin activator injections can both be lethal in minutes [7,101]. The insertion was also searched against possible mobile DNA elements, but no matches were found. Our results should be followed in the future by detailed pharmacological studies.

Conservation genetics

The low variation that exists between the solenodon sequences is hardly surprising, because the theoretical consensus in conservation genetics predicts that small populations lose genetic diversity more rapidly than large populations [102], and measures of genetic diversity have been explicitly suggested to IUCN as a factor to consider in identifying species of conservation concern [103]. The historical N_e for each subspecies was examined by our analysis (**Figure 9**), and showed lower levels recently in *S. p. woodi*. Due to the limitations of PSMC, the most recent *Ne* cannot be calculated from the genome sequences [86]. Therefore, this estimate of diversity does not reflect the recent impact on the solenodon population caused by anthropogenic factors in the last 10,000 years (**Figure 9**).

Many endangered species with small populations also have reduced heterozygosity across their genomes, and would benefit from a computational approach that reduces the cost and optimizes the amount of data for the genome assembly. The real-life scenarios where no high-quality DNA can be produced because of the remoteness of sampling location, difficulty in transportation and storage, or when the high coverage cannot be produced due to the limited funds are well known to many, especially in the field of conservation genetics. The difficult field conditions and international regulations make it difficult to obtain samples with high molecular weight DNA. To aid future conservation studies, we have mined the current dataset for microsatellite markers that are useful within and between subspecies, to be used as tools for studies on population diversity, censoring and monitoring.

The comparative analysis of the number and the length of microsatellite alleles pointed once more to the advantage of assembly B over A and C. The average length of microsatellite short tandem repeats in assembly B was the highest: 20.95 (assembly A), vs. 21.14 (assembly B) vs. 18.86 (assembly C). This may be a direct consequence of the high number of microsatellite alleles that were successfully genotyped in all of the southern samples for assembly B (2,660), as well as microsatellites that proved variable between the two subspecies but fixed within the southern samples (639). The low number of variable microsatellites between the two subspecies was likely due to the reduced amount of information obtainable from a single low coverage genome of the northern subspecies (*S. p. paradoxus*) used in this study. Venn diagrams showing overlap in microsatellite variation in three assemblies are presented in **Figure 11.**

Recently, a genetic survey using mitochondrial cytochrome b and control region sequences from 34 solenodon samples identified distinct haplotypes in northern and southern Hispaniola [16], along with a distinctive third group, a small remnant population at the Massif de la Hotte in the extreme western tip of Haiti [16,104] not sampled for this study. The north–south subspecies subdivision within *S. paradoxus* was further supported by mitogenomic sequences [12]. The island

of Hispaniola has been divided into three main biogeographic regions that differ in climate and habitat. The north and center of the island provide the largest area with known solenodon populations, and shows no discontinuity with the southeast. However, the solenodon populations in the southwestern part of the island are currently geographically isolated by Cordillera Central, and may have been isolated in the past by the ancient marine divide across the Neiba Valley (**Figure 2**). This geographic isolation is likely the reason why the *S. p. paradoxus* in the larger northern area, and *S. p. woodi* in the southwest, show morphological differences suggestive of separate subspecies [15]. Future conservation strategies directed at protecting and restoring solenodon populations on Hispaniola should take into consideration this subdivision, and treat the two subspecies as two separate conservation units.

Methods

Provenance of the samples is shown on the map (**Figure 2**), with coordinates listed in **Table S4**. Solenodons were caught with help of local guides (Nicolás Corona and Yimell Corona). During the day, potential locations were inspected in daylight for animal tracks, burrows, droppings and other signs of solenodon activity. At dawn, ambushes were set up in the forested areas along the potential animal trails. The approaching solenodons were identified by sound, and chased with flashlights when approached. Since solenodons move slowly, animals were picked up by their tails, which is the only way to avoid potentially venomous bites. All wild caught animals were released back into their habitats within 10 minutes after their capture. Before the release, the animals' tails were marked with a Sharpie pen to avoid recapturing.

Blood was drawn by a licensed ZooDom veterinarian (Adrell Núñez) from the *vena jugularis* using a 3mL syringe with a 23G x 1" needle. The blood volume collected never exceeded 1% of body weight of animals. Before the draw, an aseptic technique was applied using a povidone–iodine solution, followed by isopropyl alcohol. Once collected, the samples were transferred to a collection tube with anticoagulant (BD Microtainer, 1.0mg K2EDTA for 250–500lL volume). Collection tubes were refrigerated and transported to the lab at the *Instituto Tecnológico de Santo Domingo* (INTEC) where DNA was extracted from samples using the DNeasy Blood & Tissue kit (Qiagen, Hilden, Germany). This study has been reviewed and approved by the Institutional Animal Care and Use Committee of the University of Puerto Rico at Mayagüez (UPR-M). All the required collection and permits had been obtained before any field work was started. The samples have been collected and exported in compliance with Export Permit No: VAPB-00909 (Dominican Republic Environment and Natural Resources Ministry Viceministry of Protected Areas and Biodiversity Department of Biodiversity) and imported in compliance with CITES/ESA Import permit number 14US84465A/9 (University of Illinois Board of Trustees).

Sequencing

Sequences for *S. p. woodi* were generated by Illumina HiSeq 2000 (Illumina Inc) with 100bp paired-end reads. The Illumina HiSeq generated raw images utilizing HCS (HiSeq Control Software v2.2.38) for system control and base calling through an integrated primary analysis software called RTA (Real Time Analysis. v1.18.61.0). The BCL (base calls) binaries were converted into FASTQ utilizing the Illumina package bcl2fastq (v1.8.4). Sequences for *S. p. paradoxus* were generated by the Illumina MiSeq V3 (Illumina Inc.) at the Roy J. Carver Biotechnology Center, University of Illinois. The sequencing data for each sample used in this study is presented in **Table S5**.

Availability of supporting data and materials

Database S1: Lists of repeats in the solenodon genome (assemblies A and B)
http://public.dobzhanskycenter.ru/solenodon/repeats/solpar-a.txt
http://public.dobzhanskycenter.ru/solenodon/repeats/solpar-b.txt

Database S2: List of protein coding genes in the solenodon genome (assembly B)
http://public.dobzhanskycenter.ru/solenodon/genes/solpar-b.gff
also cds for each gene and translated sequences

Database S3: List of the annotated non-coding RNAs in the solenodon genome
http://public.dobzhanskycenter.ru/solenodon/rna

Database S5: List of single-copy orthologs in the solenodon genome (columns include: ENOG id, gene name) http://public.dobzhanskycenter.ru/solenodon/monoorthologs.txt

Database S6: List of genes with dN/dS values and GO annotations
http://public.dobzhanskycenter.ru/solenodon/selection.xls

Database S7: List of venom genes
http://public.dobzhanskycenter.ru/solenodon/venom_genes_HitGeneDB.fasta

Datablase S8: Microsatellite loci discovered in genomes of two solenodon subspecies *Solenodon paradoxus paradoxus* (northern) and *S. p. woodi* (southern), alleles, 600bp flanking regions (a total of 1,200 bp per locus), and frequency information for the two subspecies
http://public.dobzhanskycenter.ru/solenodon/STRs.xlsx

Database S9: Lists of single nucleotide differences (SND) from the assembled individual genome of Spa-1 (from *Solenodon paradoxus paradoxus*) and Spa K, - L, - M , -N, and –O (from the five *S. p. woddi*)) used to show estimates of heterozygosity in Figure 8 (see explanation in text)
http://public.dobzhanskycenter.ru/solenodon/variants

Supporting raw data is in the NCBI SRA [ENA: NKTL01000000, PROJECT: PRJNA368679] and genome assemblies, custom codes and annotations are in the *GigaScience* GigaDB database [21].

Abbreviations

bp: base pair; BCL: base call; BUSCO: Benchmarking universal single-copy orthologs; CDS: coding sequence; CEGMA: Core eukaryotic genes mapping approach; Gb: Giga base; GO: gene ontology; HMM: hidden Markov model; IUCN: International Union for Conservation of Nature; kb: kilo base; Kya: thousand (kilo) years ago; MYA: million years ago; PSMC: Pairwise Sequentially Markovian Coalescent; SND: single nucleotide difference; SNP: Single Nucleotide Polymorphism; SNV: Single Nucleotide Variation; SRA: Sequence Read Archive; STR: short tandem repeat; VCF: Variant Call Format.

Declarations

Authors have declared that they do not have any competing interests.

Endnotes

The authors thank Nicolás Corona and Yimell Corona for assistance in collecting samples. A special thanks to Kara Fore for helping to edit this manuscript. Authors at the University of Puerto Rico t Mayaguez were supported in part by NSF award #1432092. Authors at the Theodosius

Dobzhansky Center for Genome Bioinformatics were supported by the Russian Ministry of Science Mega-grant (no 11.G34.31.0068), and St. Petersburg State University grant (no 1.50.1623.2013). Sample collection in Dominican Republic was performed under permit number 00201171139 from the Ministry of Environment and Natural Resources.

References

1. MacPhee RDE, Flemming C, Lunde DP. “ Last occurrence” of the Antillean insectivoran Nesophontes: new radiometric dates and their interpretation. American Museum novitates; no. 3261. New York, NY: American Museum of Natural History; 1999;

2. Ottenwalder JA. Systematics and biogeography of the West Indian genus Solenodon. Biogeogr. West Indies Patterns Perspect. Second Ed. CRC Press; 2001. p. 253–329.

3. Roca AL, Bar-Gal GK, Eizirik E, Helgen KM, Maria R, Springer MS, et al. Mesozoic origin for West Indian insectivores. Nature. Nature Publishing Group; 2004;429:649–51.

4. Verill AH. Notes on the Habits and External Characters of the Solenodon of San Domingo (Solenodon paradoxus). Am. J. Sci. 1907;XXIV:55–7.

5. Allen JA. Notes on Solenodon paradoxus Brandt. Bull. Am. Museum Nat. Hist. 1908;XXIV:505–5017.

6. Brandt JF. De Solenodonte: novo mammalium insectivororum genere. Mem. l’Académie Impériale des Sci. St. Pétersbg. l’Académie Impériale des Sciences de St. Pétersbourg; 1833;2:459–78.

7. Derbridge JJ, Posthumus EE, Chen HL, Koprowski JL. Solenodon paradoxus (Soricomorpha: Solenodontidae). BioOne; 2015;

8. Feldhamer GA. Mammalogy: adaptation, diversity, ecology. JHU Press; 2007.

9. Wible JR. On the cranial osteology of the Hispaniolan solenodon, Solenodon paradoxus Brandt, 1833 (Mammalia, Lipotyphla, Solenodontidae). Ann. Carnegie Museum. BioOne; 2008;77:321–402.

10. Folinsbee KE, Müller J, Reisz RR. Canine grooves: morphology, function, and relevance to venom. J. Vertebr. Paleontol. BioOne; 2007;27:547–51.

11. Dufton MJ. Venomous mammals. Pharmacol. Ther. Elsevier; 1992;53:199–215.

12. Brandt AL, Grigorev K, Afanador-Hernández YM, Paulino LA, Murphy WJ, Núñez A, et al. Mitogenomic sequences support a north--south subspecies subdivision within Solenodon paradoxus. Mitochondrial DNA Part A [Internet]. Taylor & Francis; 2017;28:662–70. Available from: https://www.ncbi.nlm.nih.gov/pubmed/27159724

13. Sato JJ, Ohdachi SD, Echenique-Diaz LM, Borroto-Páez R, Begué-Quiala G, Delgado-Labañino JL, et al. Molecular phylogenetic analysis of nuclear genes suggests a Cenozoic over-water dispersal origin for the Cuban solenodon. Sci. Rep. Nature Publishing Group; 2016;6.

14. Ottenwalder JA. The distribution and habitat of Solenodon in the Dominican Republic. 1985.

15. Ottenwalder JA. The systematics, biology, and conservation of Solenodon. 1991;

16. Turvey ST, Peters S, Brace S, Young RP, Crumpton N, Hansford J, et al. Independent evolutionary histories in allopatric populations of a threatened Caribbean land mammal. Divers. Distrib. Wiley Online Library; 2016;

17. Luo R, Liu B, Xie Y, Li Z, Huang W, Yuan J, et al. SOAPdenovo2: an empirically improved memory-efficient short-read de novo assembler. Gigascience. BioMed Central; 2012;1:18.

18. Li H. Exploring single-sample SNP and INDEL calling with whole-genome de novo assembly.

Bioinformatics. Oxford Univ Press; 2012;28:1838–44.

19. Boetzer M, Henkel C V, Jansen HJ, Butler D, Pirovano W. Scaffolding pre-assembled contigs using SSPACE. Bioinformatics. Oxford Univ Press; 2011;27:578–9.

20. Wang Y, Lu Y, Zhang Y, Ning Z, Li Y, Zhao Q, et al. The draft genome of the grass carp (Ctenopharyngodon idellus) provides insights into its evolution and vegetarian adaptation. Nat. Genet. Nature Research; 2015;47:625–31.

21. Grigorev K, Kliver S, Dobrynin P, Komissarov A, Wolfsberger W, Krasheninnikova K, et al. Supporting data for "Innovative assembly strategy contributes to understanding the evolution and conservation genetics of the endangered Solenodon paradoxus from the island of Hispaniola." GigaScience Database. 2018; http://dx.doi.org/10.5524/100422.

22. Starostina E, Tamazian G, Dobrynin P, O'Brien S, Komissarov A. Cookiecutter: a tool for kmer-based read filtering and extraction. bioRxiv. Cold Spring Harbor Labs Journals; 2015;24679.

23. Marçais G, Kingsford C. A fast, lock-free approach for efficient parallel counting of occurrences of k-mers. Bioinformatics. 2011;27:764–70.

24. Marçais G, Yorke JA, Zimin A. QuorUM: an error corrector for Illumina reads. PLoS One. 2015;10:e0130821.

25. Chikhi R, Medvedev P. Informed and automated k-mer size selection for genome assembly. Bioinformatics. 2013;btt310.

26. Li H, Homer N. A survey of sequence alignment algorithms for next-generation sequencing. Brief. Bioinform. 2010. p. 473–83.

27. Gurevich A, Saveliev V, Vyahhi N, Tesler G. QUAST: quality assessment tool for genome assemblies. Bioinformatics. 2013;29:1072–5.

28. Simão FA, Waterhouse RM, Ioannidis P, Kriventseva E V, Zdobnov EM. BUSCO: assessing genome assembly and annotation completeness with single-copy orthologs. Bioinformatics. 2015;btv351.

29. Parra G, Bradnam K, Korf I. CEGMA: a pipeline to accurately annotate core genes in eukaryotic genomes. Bioinformatics. 2007;23:1061–7.

30. Hunt M, Kikuchi T, Sanders M, Newbold C, Berriman M, Otto TD. REAPR: a universal tool for genome assembly evaluation. Genome Biol. 2013;14:R47.

31. Paten B, Earl D, Nguyen N, Diekhans M, Zerbino D, Haussler D. Cactus: Algorithms for genome multiple sequence alignment. Genome Res. 2011;21:1512–28.

32. Kolmogorov M, Raney B, Paten B, Pham S. Ragout—a reference-assisted assembly tool for bacterial genomes. Bioinformatics. 2014;30:i302--i309.

33. Smit AFA, Hubley R, Green P. RepeatMasker Open-3.0. 1996.

34. Bao W, Kojima KK, Kohany O. Repbase Update, a database of repetitive elements in eukaryotic genomes. Mob. DNA. 2015;6:11.

35. Slater GSC, Birney E. Automated generation of heuristics for biological sequence comparison. BMC Bioinformatics. 2005;6:31.

36. Stanke M, Keller O, Gunduz I, Hayes A, Waack S, Morgenstern B. AUGUSTUS: ab initio prediction of alternative transcripts. Nucleic Acids Res. 2006;34:W435--W439.

37. Finn RD, Clements J, Eddy SR. HMMER web server: interactive sequence similarity searching. Nucleic Acids Res. Oxford Univ Press; 2011;gkr367.

38. Altschul SF, Gish W, Miller W, Myers EW, Lipman DJ. Basic local alignment search tool. J. Mol. Biol. Elsevier; 1990;215:403–10.

39. Bateman A, Coin L, Durbin R, Finn RD, Hollich V, Griffiths-Jones S, et al. The Pfam protein families database. Nucleic Acids Res. 2004;32:D138--D141.

40. Consortium U, others. UniProt: a hub for protein information. Nucleic Acids Res. Oxford Univ Press; 2014;gku989.

41. Nawrocki EP, Eddy SR. Infernal 1.1: 100-fold faster RNA homology searches. Bioinformatics. Oxford Univ Press; 2013;29:2933–5.

42. Nawrocki EP, Burge SW, Bateman A, Daub J, Eberhardt RY, Eddy SR, et al. Rfam 12.0: updates to the RNA families database. Nucleic Acids Res. 2014;gku1063.

43. Lowe TM, Eddy SR. tRNAscan-SE: a program for improved detection of transfer RNA genes in genomic sequence. Nucleic Acids Res. 1997;25:955–64.

44. Seemann T, Booth T. BARNAP: BAsic Rapid Ribosomal RNA Predictor [Internet]. Berlin: GitHub; 2013. p. https://github.com/tseemann/barrnap. Accessed 1st March 2018

45. Ruan J, Li H, Chen Z, Coghlan A, Coin LJM, Guo Y, et al. TreeFam: 2008 update. Nucleic Acids Res. 2008;36:D735--D740.

46. Li H, Coghlan A, Ruan J, Coin LJ, Heriche J-K, Osmotherly L, et al. TreeFam: a curated database of phylogenetic trees of animal gene families. Nucleic Acids Res. 2006;34:D572--D580.

47. Huerta-Cepas J, Szklarczyk D, Forslund K, Cook H, Heller D, Walter MC, et al. eggNOG 4.5: a hierarchical orthology framework with improved functional annotations for eukaryotic, prokaryotic and viral sequences. Nucleic Acids Res. 2015;gkv1248.

48. Stamatakis A. RAxML version 8: a tool for phylogenetic analysis and post-analysis of large phylogenies. Bioinformatics. 2014;30:1312–3.

49. Yang Z. PAML 4: phylogenetic analysis by maximum likelihood. Mol. Biol. Evol. SMBE; 2007;24:1586–91.

50. Xia X, Xie Z, Salemi M, Chen L, Wang. Y. An index of substitution saturation and its application. Mol. Phylogenet. Evol. 2003;1–7.

51. Xia X, Lemey P. Assessing substitution saturation with DAMBE. In: Philippe Lemey, Marco Salemi and Anne-Mieke Vandamme eds., editor. Phylogenetic Handb. A Pract. Approach to DNA Protein Phylogeny. 2nd ed. Cambridge Univ Press; 2009. p. 615–30.

52. Xia X. DAMBE6: New tools for microbial genomics, phylogenetics and molecular evolution. J. Hered. 2017;108:431–7.

53. Ksepka DT, Parham JF, Allman JF, Benton MJ, Carrano MT, Cranston KA, et al. The fossil calibration database—a new resource for divergence dating. Syst. Biol. 2015;syv025.

54. Benton MJ, Donoghue PCJ, Asher RJ, Friedman M, Near TJ, Vinther J. Constraints on the timescale of animal evolutionary history. Palaeontol. Electron. Paleontological Society; 2015;18:1–106.

55. Munthe K. Canidae, p. 124--143. Evol. Tert. Mamm. North Am. Cambridge Univ. Press. Cambridge. 1998;

56. Wang X, Whistler DP, Takeuchi GT. A new basal skunk Martinogale (Carnivora, Mephitinae) from late Miocene Dove Spring Formation, California, and origin of new world mephitines. J. Vertebr. Paleontol. BioOne; 2005;25:936–49.

57. Rambaut A. FigTree [Internet]. 2016. Available from: http://tree.bio.ed.ac.uk/software/figtree/ Accessed 1st March 2018

58. Springer MS, Murphy WJ, Roca AL. Appropriate fossil calibrations and tree constraints uphold the Mesozoic divergence of solenodons from other extant mammals. Mol. Phylogenet. Evol. 2018;(in press).

59. Hedges SB. Vicariance and Dispersal in Caribbean Biogeography. Herpetologica. 1996;52:466–73.

60. McDowell SB. The Greater Antillean insectivores. Bull. Am. Museum Nat. Hist. [Internet]. 1958;115:117. Available from: http://hdl.handle.net/2246/1199

61. Butler PM. Phylogeny of the insectivores. In: Benton MJ, editor. Phylogeny Classif. Tetrapods. Oxford: Clarendon; 1988. p. 117–41.

62. MacPhee RD., Novacek M. Definition and relationships of the Lipotyphla. In: Soule F, Novacek M, McKenna M, editors. Mamm. Phylogeny, Vol. 2, Placentals. New York: Springer-Verlag; 1993. p. 13–31.

63. McKenna M, Bell S, Simpson S. Classification of mammals above the species level. New York: Columbia University Press; 1997.

64. Edgar RC. MUSCLE: multiple sequence alignment with high accuracy and high throughput. Nucleic Acids Res. 2004;32:1792–7.

65. Suyama M, Torrents D, Bork P. PAL2NAL: robust conversion of protein sequence alignments into the corresponding codon alignments. Nucleic Acids Res. 2006;34:W609--W612.

66. Soto-Girón MJ, Ospina OE, Massey SE. Elevated levels of adaption in Helicobacter pylori genomes from Japan; a link to higher incidences of gastric cancer? Evol. Med. public Heal. 2015;eov005.

67. Gharib WH, Robinson-Rechavi M. The branch-site test of positive selection is surprisingly robust but lacks power under synonymous substitution saturation and variation in GC. Mol. Biol. Evol. SMBE; 2013;mst062.

68. Tang H, Klopfenstein D, Pedersen B, Flick P, Sato K, Ramirez F, et al. GOATOOLS: Tools for Gene Ontology [Internet]. Zenodo; 2015. Available from: https://doi.org/10.5281/zenodo.31628 Accessed 1st March 2018

69. Baggs JE, Green CB. Nocturnin, a deadenylase in Xenopus laevis retina: a mechanism for posttranscriptional control of circadian-related mRNA. Curr. Biol. 2003;13:189–98.

70. Oleksyk TK, Smith MW, O'Brien SJ. Genome-wide scans for footprints of natural selection. Philos. Trans. R. Soc. London Ser. B Biol. Sci. 2010;365:185–205.

71. Fay JC, Wyckoff GJ, Wu CI. Positive and negative selection on the human genome. Genetics 2001;158:1227–34.

72. Fay JC, Wu C-I. The Neutral Theory in the Genomic Era. Curr. Opin. Genet. Dev. 2001;11:642–6.

73. Ellegren H. Evolution: Natural selection in the evolution of humans and chimps. Curr. Biol. 2005.

74. Gayà-Vidal M, Albà M. Uncovering adaptive evolution in the human lineage. BMC Genomics 2014;15:599.

75. Bakewell MA, Shi P, Zhang J. More genes underwent positive selection in chimpanzee evolution than in human evolution. Proc. Natl. Acad. Sci. 2007;104:7489–94.

76. Olson M V., Varki A. Sequencing the chimpanzee genome: insights into human evolution and disease. Nat. Rev. Genet. 2003;4:20–8. doi:10.1038/nrg981

77. Mi H, Huang X, Muruganujan A, Tang H, Mills C, Kang D, et al. PANTHER version 11: expanded annotation data from Gene Ontology and Reactome pathways, and data analysis tool enhancements. Nucleic Acids Res. 2016;gkw1138.

78. Jungo F, Bougueleret L, Xenarios I, Poux S. The UniProtKB/Swiss-Prot Tox-Prot program: a central hub of integrated venom protein data. Toxicon 2012;60:551–7.

79. Langmead B, Salzberg SL. Fast gapped-read alignment with Bowtie 2. Nat. Methods. 2012;9:357–9.

80. Li H, Durbin R. Fast and accurate short read alignment with Burrows-Wheeler transform. Bioinformatics. 2009;25:1754–60.

81. Danecek P, Auton A, Abecasis G, Albers CA, Banks E, DePristo MA, et al. The variant call format and VCFtools. Bioinformatics. 2011;27:2156–8.

82. Dobrynin P, Liu S, Tamazian G, Xiong Z, Yurchenko AA, Krasheninnikova K, et al. Genomic legacy of the African cheetah, Acinonyx jubatus. Genome Biol. 2015. 16:277. doi: 10.1186/s13059-015-0837-4..

83. Gordon D, Huddleston J, Chaisson MJP, Hill CM, Kronenberg ZN, Munson KM, et al. Long-read sequence assembly of the gorilla genome. Science (80-.). 2016;352:aae0344-aae0344. doi:10.1126/science.aae0344

84. Li R, Fan W, Tian G, Zhu H, He L, Cai J, et al. The sequence and de novo assembly of the giant panda genome. Nature. 2010;463:1106–1106. doi:10.1038/nature08846

85. Cho YS, Hu L, Hou H, Lee H, Xu J, Kwon S, et al. The tiger genome and comparative analysis with lion and snow leopard genomes. Nat. Commun. 2013;4.doi:10.1038/ncomms3433

86. Li H, Durbin R. Inference of human population history from individual whole-genome sequences. Nature. 2011;475:493–6.

87. Weber JL. Human DNA polymorphisms and methods of analysis. Curr. Opin. Biotechnol. 1990;1:166–71.

88. Weber JL, Wong C. Mutation of human short tandem repeats. Hum. Mol. Genet. 1993;2:1123–8.

89. Weber JL, May PE. Abundant class of human DNA polymorphisms which can be typed using the polymerase chain reaction. Am. J. Hum. Genet. 1989;44:388–96. PMC1715443

90. Benson G. Tandem repeats finder: a program to analyze DNA sequences. Nucleic Acids Res. 1999;27:573–80.

91. Willems T, Zielinski D, Yuan J, Gordon A, Gymrek M, Erlich Y. Genome-wide profiling of heritable and de novo STR variations. Nat Methods. 2017 Jun;14(6):590-592. doi: 10.1038/nmeth.4267.

92. Koepfli K-P, Paten B, O'Brien SJ. The Genome 10K Project: a way forward. Annu. Rev. Anim. Biosci. Annual Reviews; 2015;3:57–111.

93. Compeau PEC, Pevzner PA, Tesler G. How to apply de Bruijn graphs to genome assembly. Nat. Biotechnol. 2011;29:987–91.

94. Myers EW. Toward simplifying and accurately formulating fragment assembly. J. Comput. Biol. 1995;2:275–90.

95. Myers EW. The fragment assembly string graph. Bioinformatics. 2005;21:ii79--ii85.

96. Bradnam KR, Fass JN, Alexandrov A, Baranay P, Bechner M, Birol I, et al. Assemblathon 2: evaluating de novo methods of genome assembly in three vertebrate species. Gigascience. 2013;2:10.

97. Gogarten JP, Olendzenski L. Orthologs, paralogs and genome comparisons. Curr. Opin. Genet. Dev. 1999. p. 630–6.

98. Creevey CJ, Muller J, Doerks T, Thompson JD, Arendt D, Bork P. Identifying Single Copy Orthologs in Metazoa. PLOS Comput. Biol. 2011;7:e1002269.

99. Treangen TJ, Salzberg SL. Repetitive DNA and next-generation sequencing: computational challenges and solutions. Nat. Rev. Genet. 2012;13:36–46.

100. Volfovsky N, Oleksyk TK, Cruz KC, Truelove AL, Stephens RM, Smith MW. Genome and gene alterations by insertions and deletions in the evolution of human and chimpanzee chromosome 22. BMC Genomics. 2009;10:51.

101. Rabb GB. Toxic salivary glands in the primitive insectivore Solenodon. Nat. Hist. Misc. 1959;170:1–3.

102. Allendorf FW, Luikart G. Conservation and the genetics of populations. John Wiley & Sons; 2009.

103. Willoughby JR, Sundaram M, Wijayawardena BK, Kimble SJA, Ji Y, Fernandez NB, et al. The reduction of genetic diversity in threatened vertebrates and new recommendations regarding IUCN conservation rankings. Biol. Conserv. 2015;191:495–503.

104. Turvey ST, Meredith HMR, Scofield RP. Continued survival of Hispaniolan solenodon Solenodon paradoxus in Haiti. Oryx. Cambridge Univ Press; 2008;42:611–4.

105. Consortium GO, others. The Gene Ontology (GO) database and informatics resource. Nucleic Acids Res. Oxford Univ Press; 2004;32:D258--D261.

Tables

Table 1. Description of the assembly strategies and comparison of metrics for the resulting assemblies

Assembly Names	A	B	C	D
**Assembly Tools**				
Contig assembly tool	*Fermi*	*Fermi*	*SOAPdenovo2*	*SOAPdenovo2*
Scaffolding tool	*SOAPdenovo2*	*SSPACE*	*SOAPdenovo2*	*SSPACE*
Gap closing tool	*GapCloser*	*GapCloser*	*GapCloser*	*GapCloser*
**Assembly Metrics**				
**Total contigs (>1,000 bp)**	**71,429**	**71,429**	**189,566**	**189,566**
Contig N50	54,944	54,944	4,048	4,048
Contig CEGMA (%) *	96.37(77.42)	96.37(77.42)	68.15(33.06)	68.15(33.06)
Contig BUSCO (%)	86(65)	86(65)	42(21)	42(21)
**Total scaffolds (>1,000 bp)**	**14,417**	**40,372**	**20,466**	**-**
Final N50	555,585	110,915	331,639	-
Final CEGMA (%)	95.56(81.85)	95.97(88.71)	95.97(90.73)	-
Final BUSCO (%)	91(74)	86(64)	94(80)	-
**Quality**				
Percentage of *N*s (%)	0.06322	0.0135	0.02622	-
REAPR error-free bases (%)	96.46	95.35	94.98	-
REAPR low-scoring regions	18	16	71	-
REAPR incorrectly oriented reads	11,543	5,329	28,964	-

* BUSCO [28] and CEGMA [29] percentages are reported for all genes (complete and partial), while the percentage of complete genes are shown in parentheses.

Table 2. Pairwise genomic coverage for the three assemblies and the *Sorex araneus* genome (SorAra 2.0, NCBI accession number GCA_000181275.2) obtained from the *Progressive Cactus* [31] alignments. While all three assemblies have similar amounts of syntenic coverage to the *Sorex* genome, assembly B contains the least numbers of structural rearrangements (inversions and translocations) compared to the other two assemblies (A and C).

		vs S. paradoxus woodi			*vs S. araneus*		
		Pairwise genome coverage (%) *				# Inversions	# Translocations
	Assembly	A	B	C			
S. araneus		42.1	42.2	42.3	-	-	-
	A	-	99.4	98.5	35.5	87	5
S. p. woodi	B	99.3	-	99.3	35.5	34	0
	C	98.4	98.5	-	35.5	81	2

* Values in cells at the intersection of rows and columns represent the percentage (%) of coverage between the two compared genome assemblies. Syntenic blocks between each of the three solenodon assemblies (A, B and C) were compared to the *S. araneus* assembly, and 50Kbp syntenic blocks were identified using the *ragout-maf2synteny* module of the software package Ragout [32].

Table 3. Repeat content of the *Solenodon paradoxus* genome (Assembly B), annotated by RepeatMasker [33] with the RepBase library [34].

Class		Number	Length (bp)	Percentage (%)
	**Total interspersed repeats**		**461,754,432**	**22.53**
**SINEs**		**271,839**	**36,271,455**	**1.77**
	Alu/B1	6	341	<0.0001
	MIRs	264,319	35,557,190	1.73
**LINEs**		**610,079**	**304,823,409**	**14.87**
	LINE1	425,750	260,176,709	12.7
	LINE2	157,422	39,432,276	1.92
	L3/CR1	22,172	4,293,335	0.21
	RTE	4,122	839,744	0.04
**LTR elements**		**246,305**	**78,108,726**	**3.81**
	ERVL	61,150	24,158,692	1.18
	ERVL-MaLRs	94,934	30,075,905	1,47
	ERV_classI	57,674	19,259,649	0.94
	ERV_classII	24,454	2,840,874	0,14
**DNA elements**		**204,413**	**42,015,054**	**2.05**
	hAT-Charlie	112,664	21,168,194	1.03
	TcMar-Tigger	43,950	11,141,107	0.54
**Small RNAs**		**4,772**	**456,810**	**0.02**
**Satellites**		**46,734**	**20,910,815**	**1.02**
**Simple repeats**		**644,811**	**28,549,871**	**1.39**
**Low complexity regions**		**114,188**	**5,933,786**	**0.29**
**Unclassified**		**3,051**	**535,788**	**0.03**

Table 4. The weighted coverages of the genomes in the Progressive Cactus alignment [31], as calculated against the *C. familiaris* genome. The weighted coverage of the *S. paradoxus* genome assembly from our study is comparable to other high coverage mammalian genome assemblies. The cladogram used for multiple genome alignment with Progressive Cactus is shown in **Figure S1**.

Query genome	Weighted coverage
Dog (*Canis familiaris*)	(1.14)*
Cow (*Bos taurus*)	1.06
Common shrew (*Sorex araneus*)	1.05
Star-nosed mole (*Condylura cristata*)	1.04
Hispaniolan solenodon (*Solenodon paradoxus*)	1.05

* The weighted coverage of a genome to itself is parenthesized as it is not a comparative value

Table 5. Fossil-based priors associated with mammalian evolution used for calibration of divergence times [53–56]. The 4,416 single copy orthologs identified in our assembly were used for phylogeny inference via four-fold degenerate sites with programs RAxML [48] and PAML [49]. The resulting phylogenetic tree was plotted with FigTree [57] and is presented in **Figure 5**.

**Node**	**Calibration prior on clade**	**Node *min.* age (Mya)**	**Node *max*. age (Mya)**	**Evidence**
Opossum - placental mammals split	*Eutheria - Metatheria*	157.3	169.6	Fossil (Benton et al. 2015)
Human - mouse	*Archonta - Glires*	61.5	100.5	Biostratigraphy (Benton and Donoghue, 2007)
Primates, mouse - dog, horse, cow	*Euarchontaglires - Laurasiatheria*	61.6	100.5	Fossil (Benton et al. 2015)
Dog - ferret	*Canidae - Arctoidea*	35	45	Fossil (Wang et al., 2005; Munthe, 1998)
Solenodon - hedgehog, shrew, mole	*Lipotyphla*	61.6	100.5	Fossil (Benton et al. 2015)
Cow - horse	*Artiodactyla* as soft minimum	52.4	100.5	Fossil (Benton et al. 2015)

Table 6. The putative targets of positive selection in the solenodon genome. The dN/dS values and the GO categories for the 12 genes that showed signatures of positive selection in the *Solenodon paradoxus woodi* genome (dN>dS). All other genes are reported in **Database S6**.

Solenodon gene	dS	dN	dN/dS	GO category description	Human ortholog
ENOG410UG5H	0.000003	0.002563	≥999	Plasma membrane	*KLF9*
ENOG410USMX	0.000011	0.010830	≥999	Plasma membrane	*TNFSF13B*
ENOG410UWRE	0.000015	0.014790	≥999	-	*SMIM3*
ENOG410UNED	0.000174	0.030411	174.84	-	*CCRN4L*
ENOG410UJP8	0.013214	0.120449	9.12	Cytosol	*PLK4*
ENOG410UWA9	0.020955	0.104972	5.01	Mitochondrion	*NDUFC1*
ENOG410V3Q6	0.047538	0.071112	1.50	Plasma membrane	*SYT16*
ENOG410UQAM	0.078543	0.096445	1.23	-	*WBP2NL*
ENOG410UKXY	0.168982	0.185535	1.10	-	*TIGIT*
ENOG410UKXJ	0.134581	0.146926	1.09	Cytoplasm	*LRRC66*
ENOG410UIAB	0.060622	0.065402	1.08	-	*TMEM56*
ENOG410UG23	0.176172	0.177344	1.01	Generation of precursor metabolites and energy	*THTPA*

Table 7. Homologous matches for the most relevant protein venom classes in the *Solenodon paradoxus* genome. Genes were identified by querying 6,534 toxin and venom protein representatives found in animal venoms in Tox-Prot from Uniprot [78]. All of the protein groups are present in snake venoms. The sequences of the putative venom genes from *S. paradoxus* are available in the **Database S7**.

Protein groups found in animal venoms	Number of matches in the *S. paradoxus* genome
Metalloproteinase; Serine protease	8 each
Hyaluronidase	6
(Acetyl)Cholinesterase	2
Calglandulin; Nerve growth factors	4 each
Lipase	3
Hydrolase; Kunitz serine protease inhibitor; Nucleotidase; O-methyltransferase; Oxidase; Peptidase; Phosphodiesterase; Phospholipase; Vascular endothelial growth factor	1 each

Figures

Figure 1. The two subspecies of *Solenodon paradoxus*. A) A captive Hispaniolan solenodon from the northern subspecies (*S. p. paradoxus*) photographed at the Santo Domingo Zoo (photo taken by Juan C. Martínez-Cruzado in 2014). **B).** A mounted specimen of the southern subspecies (*S. p. woodi*) photographed at the *Museo Nacional de Historia Natural prof. Eugenio de Jesús Marcano* in Santo Domingo, Dominican Republic (photo taken by Taras K. Oleksyk in 2017).

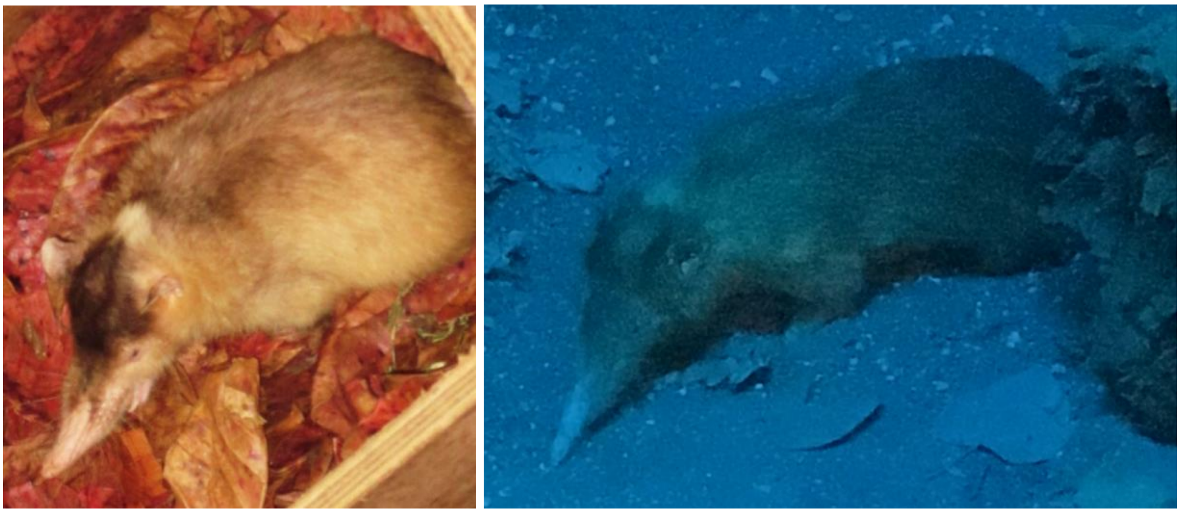

A. B.

Figure 2. Origins of the genomic DNA samples of *Solenodon paradoxus* from the island of Hispaniola. Approximate locations of capture for five wild individuals of *S. p. woodi*: Spa-K and Spa-L from La Cañada del Verraco, as well as Spa-M, Spa-N, and Spa-O from the El Manguito location in the Pedernales Province in the southwest corner of the Dominican Republic bordering Haiti. In addition, one *S. p. paradoxus* sample (Spa-1) from Cordillera Septentrional in the northern part of the island. Exact coordinates of each sample location are listed in Brand et al. 2017. The dashed line indicates the position of the Cul de Sac Plain and Neiba Valley; this region was periodically inundated by a marine canal that separated Hispaniola into north and south paleo-islands during the Pliocene and Pleistocene [15]. The original map is in the public domain (courtesy of NASA).

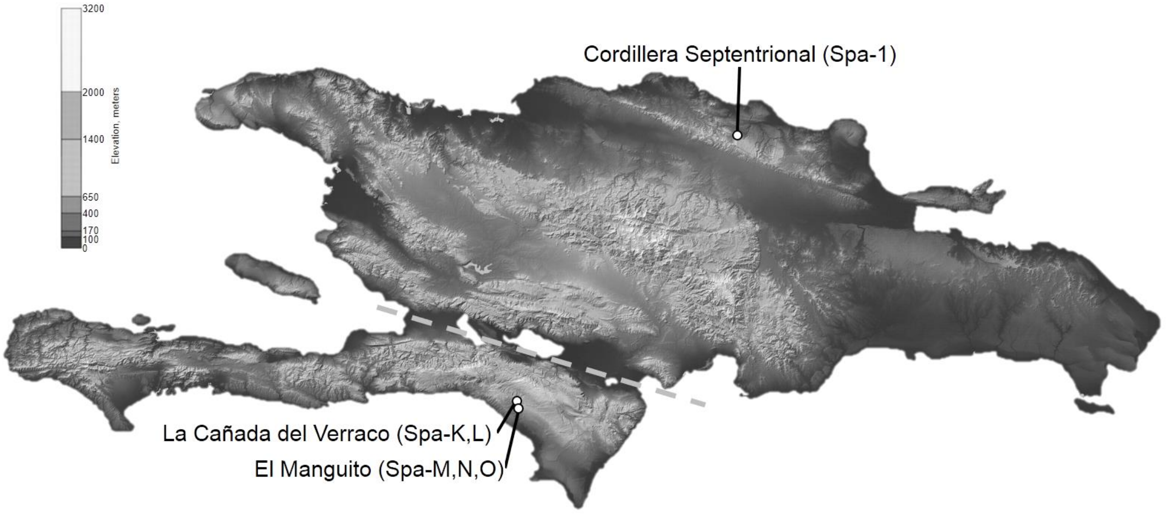

Figure 3. Heterozygosity and k-mer distribution. k-mer distributions for the *S. p. woodi* reads. Only one original sample (SPA-K) distribution is shown as a solid gray line, as the distributions were identical for each of the individual samples. The predicted mean genome coverage was approximately 5x for each sample (x=5). One example is plotted by a black solid line on the left. The combined uncorrected dataset is plotted in a dashed red line indicates a maximum at x=26. The combined dataset corrected with QuorUM [24] is plotted in a solid blue line, also with a maximum at x=26. A smaller local maximum on the left side for both combined distributions, corrected and uncorrected (representing k-mers found once or very few times) is expected from differences between overlapping reads, most likely the sequencing errors. Other local maxima (seen as a small bulge at the x=5) are interpreted as heterozygous sites. These proved to have almost no impact on the combined sample even after read correction, indicating a lack of heterozygous sites for this solenodon subspecies. The largest local maxima (to the right) are interpreted as projected coverage. For the combined samples, this value is x=26..

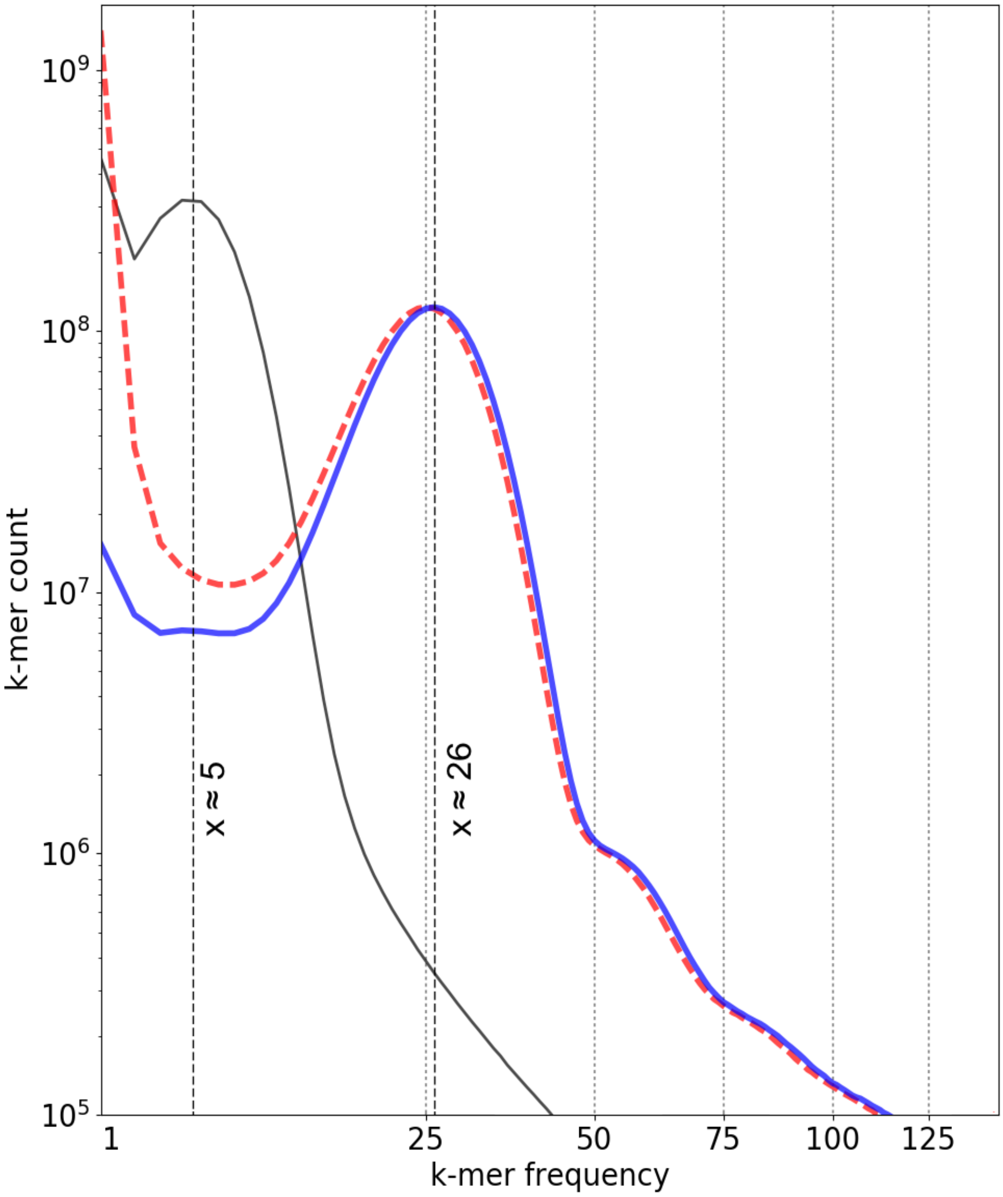

Figure 4. Distribution of the gene prediction support by extrinsic evidence for *Solenodon* assemblies A (on the left) and B (on the right). Proteins of four reference species *S. araneus* (SorAra 2.0, GCA_000181275.2), *Erinaceus europaeus* (EriEur2.0, GCA_000296755.1), *Homo sapiens* (GRCh38.p7) and *Mus musculus* (GRCm38.p4) were aligned to a *S. paradoxus* assembly with Exonerate [35] with a maximum of three best matches per protein. Coding sequences (CDS) were cut from each, clustered and uploaded into the AUGUSTUS software package [36] to predict genes in the soft-masked *Solenodon* assembly. Proteins from the predicted genes were aligned by HMMER [37] and BLAST [38] to Pfam [39] and Swiss-Prot [40] databases. Genes supported by matches to protein databases and "hints" (see definition in main text) were retained; the rest were discarded. Substantially more transcripts have higher hint support in assembly B. The annotated genes can be retrieved from **Database S2**. Assembly C has not been evaluated.

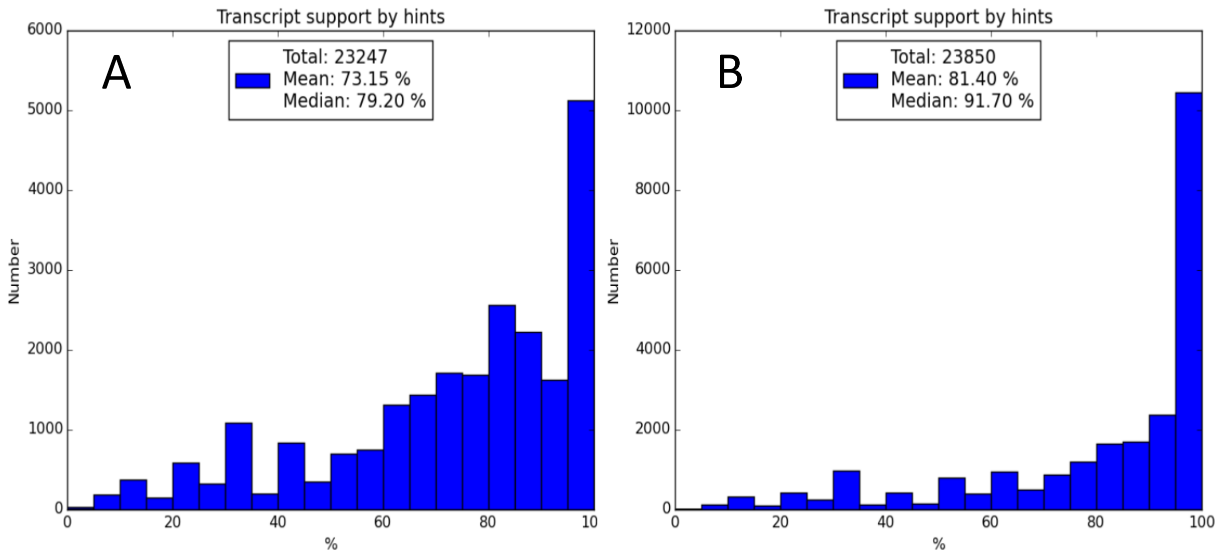

Figure 5. Phylogenetic relationships of *Solenodon paradoxus* and other mammals from whole-genome data. **A.** Maximum likelihood phylogeny showing branch lengths. The tree was built using RAxML [48] with the PROTGAMMAAUTO option and the JTT fitting model tested with 1,000 bootstrap replicates. **B.** Divergence time estimates based on 461,539 four-fold degenerate sites from the codon alignments of single-copy orthologs and using fossil-based priors (**Table 5**). The divergence time estimation was made by the MCMCtree tool from the software package PAML [49] with the HKY+G model of nucleotide substitutions and 2,200,000 generations of MCMC (of which the first 200,000 generations were discarded as burn-in). The 95% confidence intervals are given in square brackets and depicted as semitransparent boxes around the nodes. The inferred divergence time of *S. paradoxus* from other mammals is 73.6 Mya (95% confidence interval of 61.4-88.2 Mya).

A.

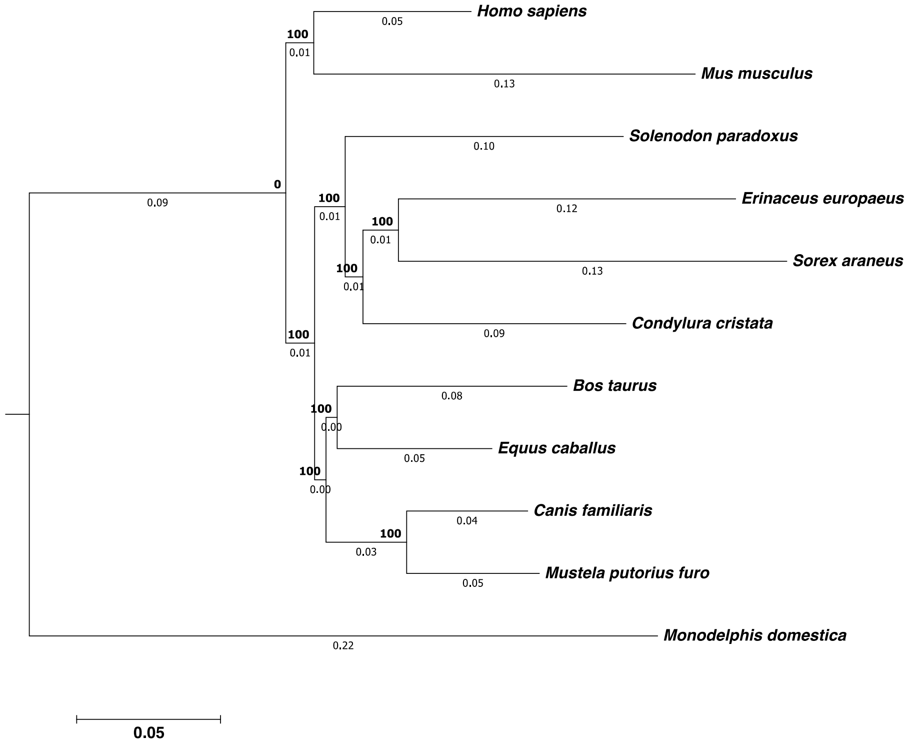

B.

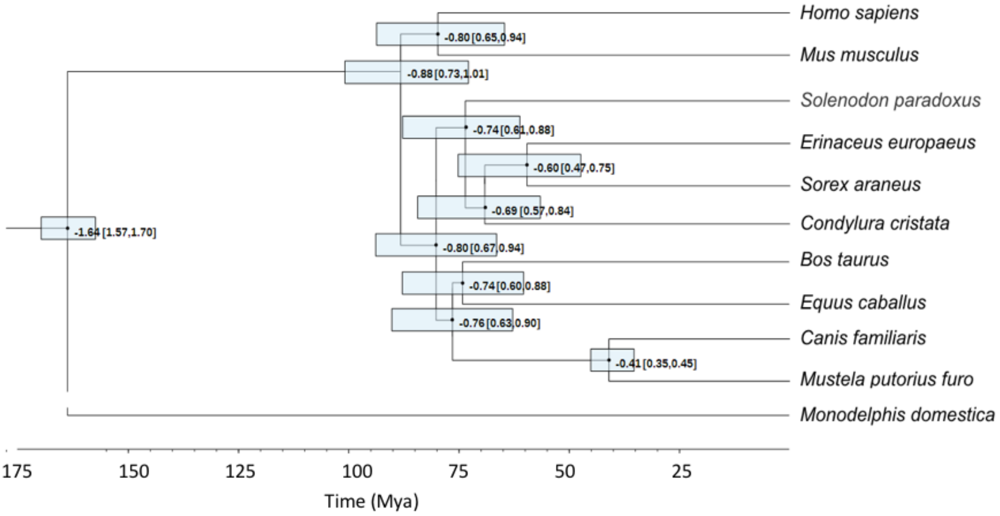

Figure 6. The dN/dS ratios for 4,416 single copy orthologous genes. The dN and dS ratios were calculated with the *codeml* module from the PAML package [49], and calculated over the entire length of a protein coding gene. Values are color-coded by GO term aggregated by the GO Slim generic database [68,105], and the color code legend is presented in **Figure S2**. The solid black line represents dN=dS; dots above it represent genes showing signatures of positive selection. The figure is truncated at dN=1 and dS=2, so larger values are not shown on the graph, but all ω, dN, and dS values are available in **Database S6.**

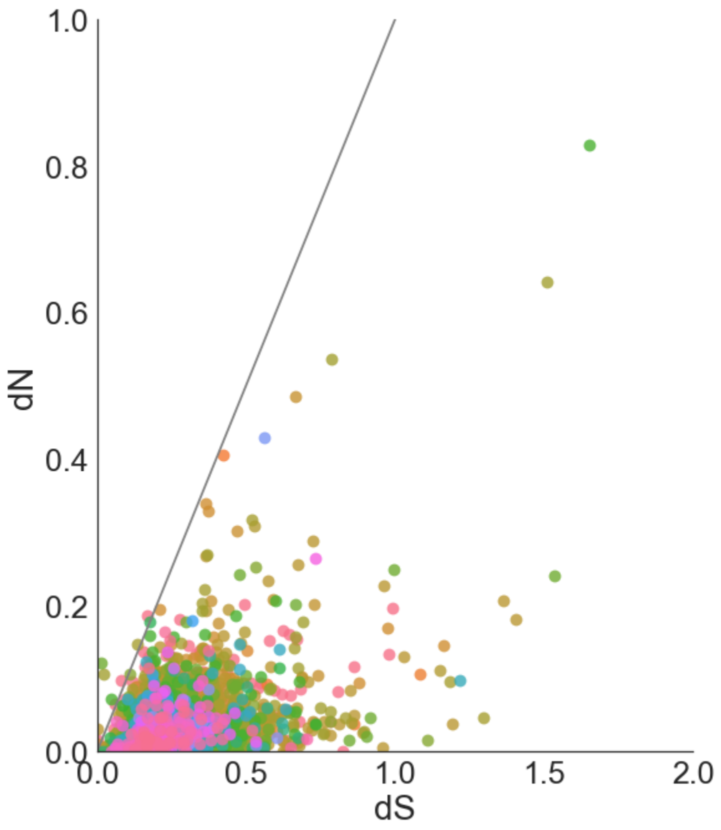

Figure 7. (A) Predicted coagulation factor X (F10) gene structure arrangement from the structure of known homologs (due to the scaffolding, the total gene length is unknown in solenodon). The 21 codon insertion is highlighted in red on exon two of the solenodon F10 gene. Exons are represented as black boxes and introns as lines connecting exons. (B) F10 protein sequence alignment showing an unusual insertion in the *Solenodon paradoxus* genome absent in all other mammalian and reptilian genes retrieved from the Tox-Prot from Uniprot [78]. The insertion of 21 amino acids is indicated with a red-boxed line in the alignment. (C) Reconstructed mammalian F10 phylogenetic maximum likelihood tree using the model GTR+I+Γ, 1000 bootstrap replicates (1590 bp-long alignment). The numbers set indicate approximate likelihood-ratio branch test (aLRT), Bayesian-like modification of the aLRT and bootstrap percentage, respectively.

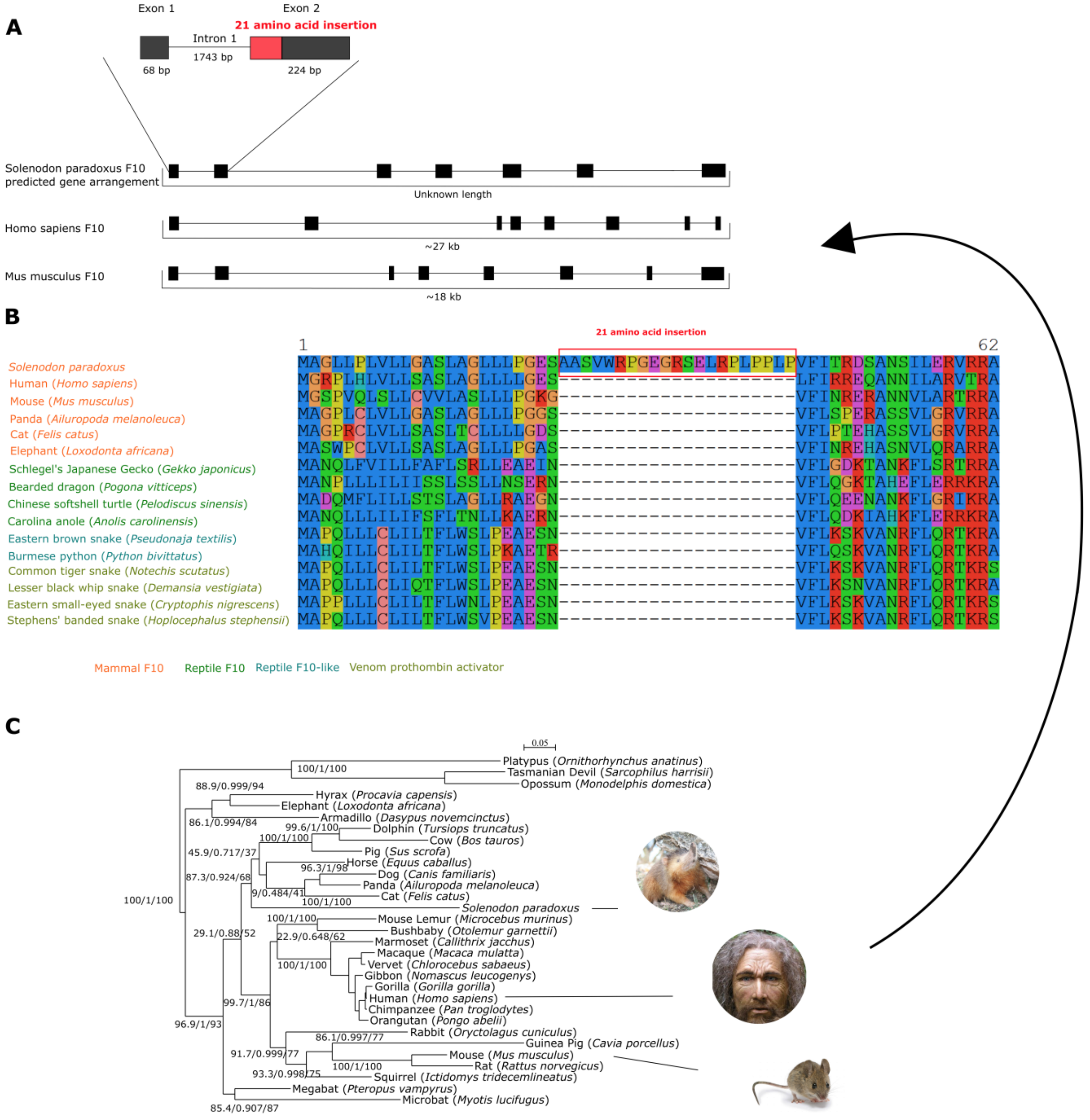

Figure 8. Low genome heterozygosity in *Solenodon paradoxus woodi* compared to other mammalian taxa. The SNV rate in the *S. p. woodi* genome is shown relative to other mammal genomes as an estimate of genome diversity (*h*). The value for each sequenced individual was estimated using all variant positions, with repetitive regions not filtered. The SNVs are deposited in **Database S9**.

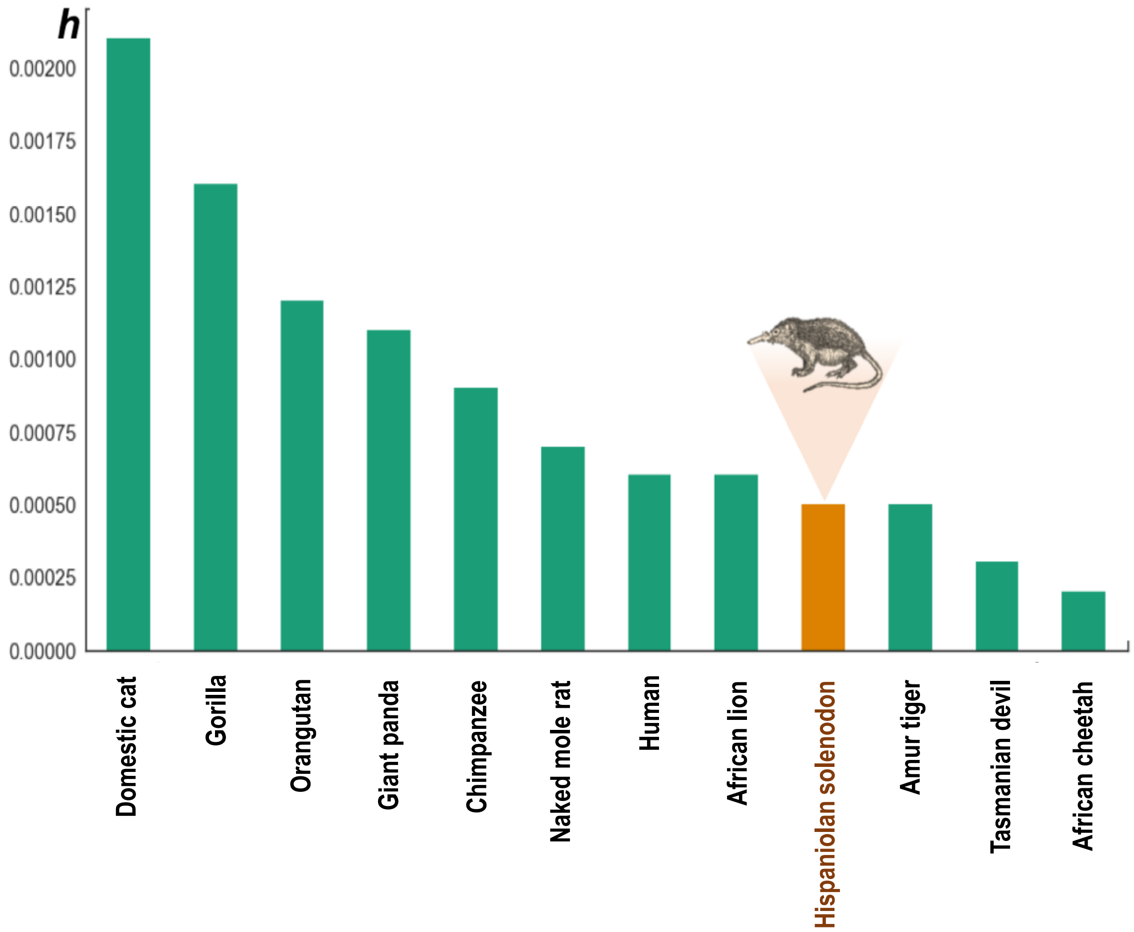

Figure 9. Demographic history inference for the southern *S. p. woodi* (red) and the northern *S. p. paradoxus* (blue) subspecies using the pairwise sequentially Markovian coalescent (PSMC) model [86].

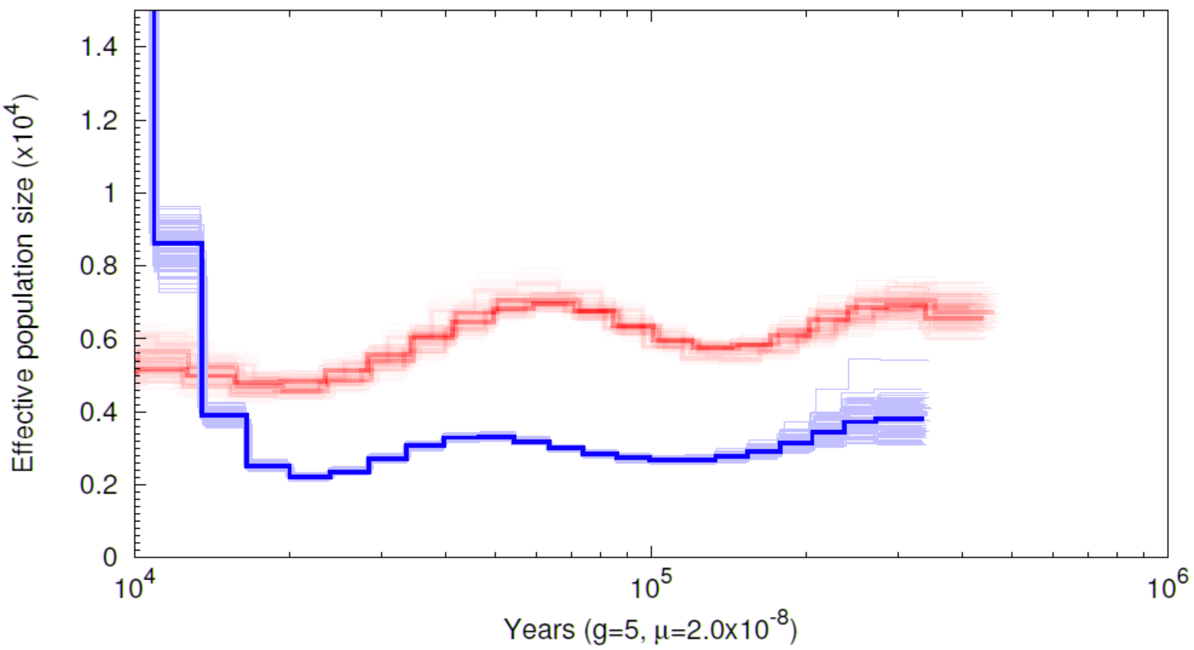

Figure 10. (A) Simplified version of the coagulation cascade, indicating key steps involving the coagulation factor X (F10). (B) Protein modeling of solenodon sequence data using SWISS-MODEL. The target model (4bxs) used was the F10-like protease of the venomous elapid snake *Pseudonaja textilis*. Due to its location the insertion cannot be represented in the model (its location is indicated according to the PDB annotation). Colors indicate model quality, with red being low quality and blue high quality modeling. Colors also separate F10's light chain (EGF-like domain) in red from the heavy chain (serine protease domain) in blue (the half circle line in black separates both domains). (C) Amino acid sequence properties calculated for the solenodon F10 translated gene, with focus on the insertion region 23-43. One signal peptide cleavage site was detected between position 25 and 26. Predicted protein interaction sites at position 26, 29-30 and 32-40. Hydropathy analysis showed a relatively hydrophilic structure for the insertion.

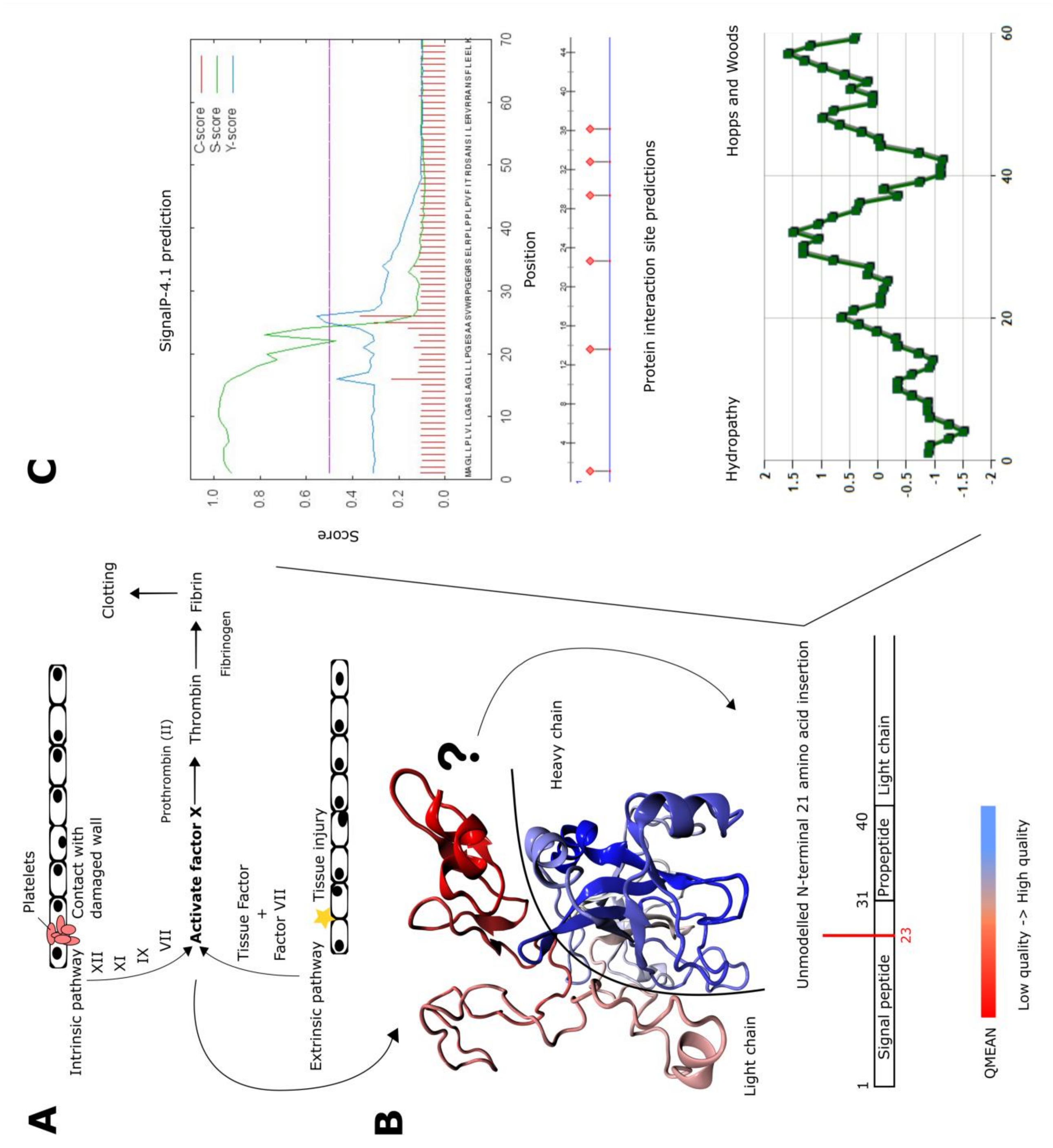
A
Intrinsic pathway
Platelets
Contact with damaged wall
XII
XI
IX
VII
Activate factor X
Prothrombin (II)
Thrombin
Fibrinogen
Fibrin
Clotting
Tissue Factor
+
Factor VII
Tissue injury
Extrinsic pathway
B
?
Heavy chain
Light chain
Unmodelled N-terminal 21 amino acid insertion
1
31
40
23
Signal peptide
Propeptide
Light chain
QMEAN
Low quality -> High quality
C
SignalP-4.1 prediction
C-score
S-score
Y-score
Score
Position
Protein interaction site predictions
Hydropathy
Hopps and Woods

Figure 11. Numbers of variable microsatellite alleles discovered in *S. paradoxus* assemblies. The diagrams were built independently for Fermi-based assemblies (A and B) and one SOAPdenovo2 based assembly (C). The red circle indicates microsatellites that were successfully genotyped in all samples with at least one alternative allele in the southern subspecies (*S. p. woodi*). The blue circle indicates microsatellites that were successfully genotyped in all samples with at least one alternative allele in the northern subspecies (*S. p. paradoxus*). The overlap indicates microsatellite loci with at least one alternative variant found in both subspecies. All alleles discovered, number of fixed alleles in each population and number of unique alleles in each population are presented in **Table S3.** All the candidate microsatellite loci discovered in this study, along with their 5' and 3' flanking regions are listed in the **Database S8.**

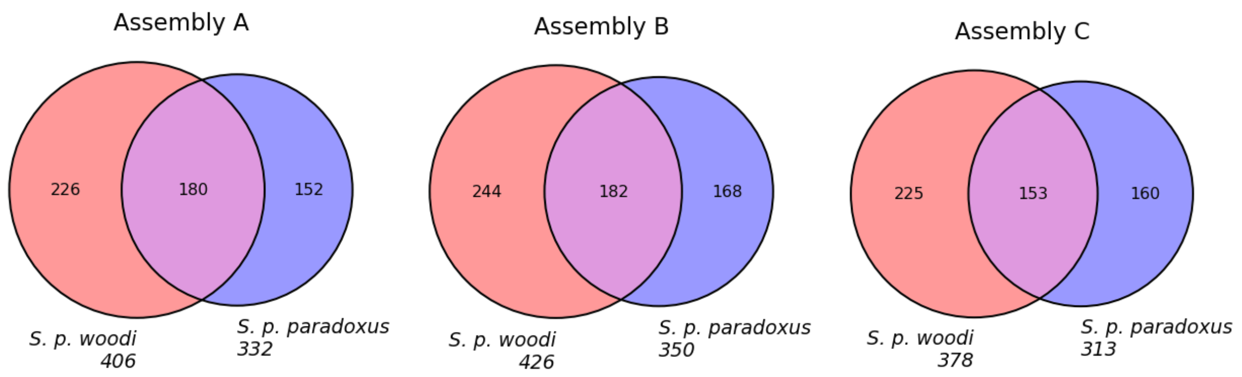

Click here to access/download
Supplementary Material
Grigorev et al. GigaScience.v.14 Supplementary Tables.docx

Supplementary Figures

Click here to access/download

Supplementary Material

Grigorev et al. GigaScience.v.14 Supplementary Figures.docx

Personal Cover - for graphical abstract

Click here to download Personal Cover Solenodon 1.1.jpg

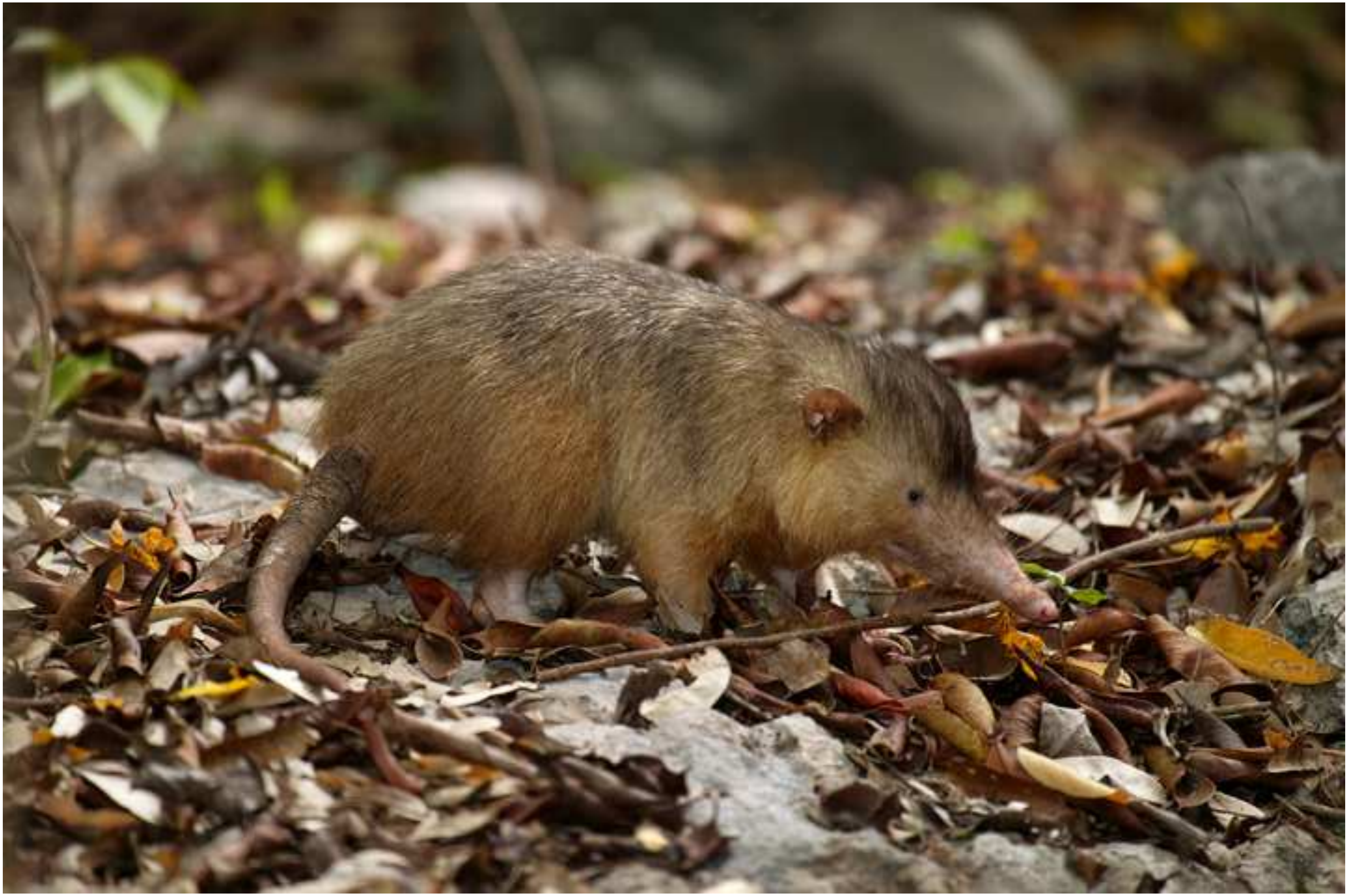

Supplement: GIGA-D-17-00182_Revision_2.pdf [file giy025_giga-d-17-00182_revision_2.pdf]
