# Supplementary material for: Innovative assembly strategy contributes to understanding the evolution and conservation genetics of the endangered Solenodon paradoxus from the island of Hispaniola
Source: Gigascience. 2018 Mar 16;7(6):giy025. doi: 10.1093/gigascience/giy025 (PMC6009670; doi:10.1093/gigascience/giy025)

**Figure S1.** The phylogenetic tree used for multiple genome alignment with Progressive Cactus


(Paten et al. 2011). The taxa have been chosen based on their availability and the quality of genome assembly, not to make inferences about mammalian phylogeny. This cladogram only shows tree topology, and the branches do not represent evolutionary time, and do not assume the basal position of *Solenodon* *paradoxus*.


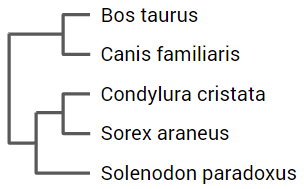


**Figure S2.** The colors assigned to GO terms represented in Figure 4.


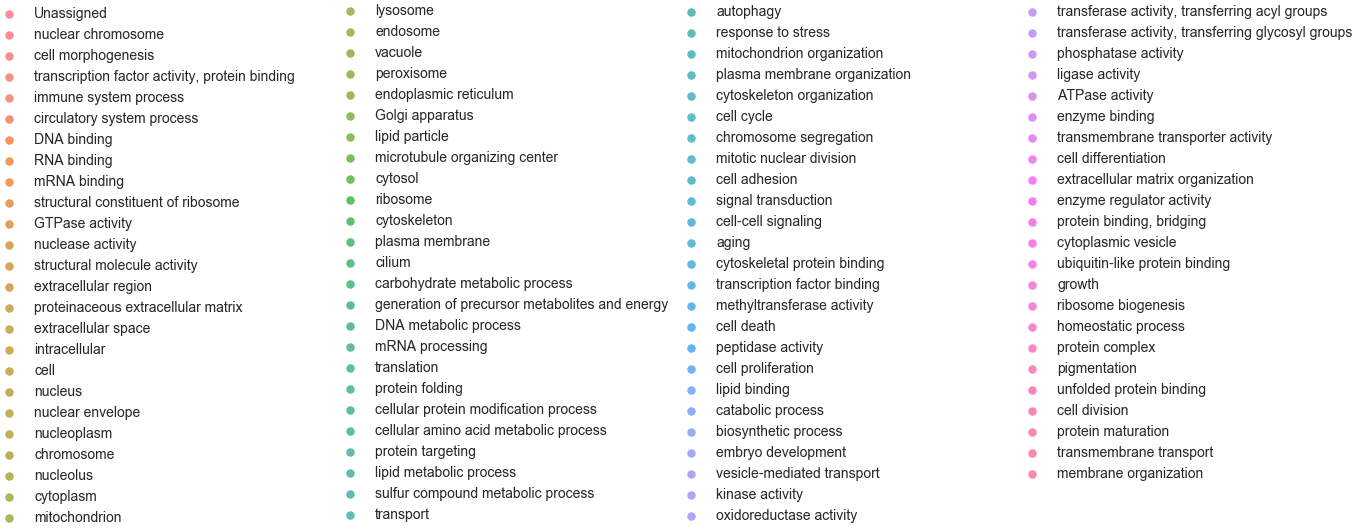

Supplement: Supplement Files [file giy025_supplement_files.zip › Grigorev et al. GigaScience.v.14 Supplementary Figures.docx]
