# Supplementary material for: Innovative assembly strategy contributes to understanding the evolution and conservation genetics of the endangered Solenodon paradoxus from the island of Hispaniola
Source: Gigascience. 2018 Mar 16;7(6):giy025. doi: 10.1093/gigascience/giy025 (PMC6009670; doi:10.1093/gigascience/giy025)
Supplement: Supplement Files [file giy025_supplement_files.zip › Grigorev et al. GigaScience.v.14 Supplementary Tables.docx]

**Table S1.** Classification of the fast-evolving genes (dN/dS>0.25) in solenodon genome using PANTHER Overrepresentation Test (release 20160715) and GO Ontology database (Released 2017-02-28) based on the *Homo sapiens* genes.  Genes are only represented if P<0.05 after the Bonferroni correction for multiple testing (Treangen and Salzberg, 2012)

|  | **# genes in reference** | **Observed #** | **Expected#** | **Fold Enrichment** | **Positive/negative** | **P value** |
| --- | --- | --- | --- | --- | --- | --- |
| **GO biological process** | | | | | | |
| inflammatory response | 456 | 36 | 14.5 | 2.48 | + | 0.00825 |
| **GO molecular function** | | | | | | |
| cytokine receptor activity | 91 | 14 | 2.89 | 4.84 | + | 0.00539 |
| hormone activity | 122 | 16 | 3.88 | 4.12 | + | 0.00784 |
| **GO cellular component** | | | | | | |
| external side of plasma membrane | 280 | 26 | 8.91 | 2.92 | + | 0.00249 |
| cell surface | 765 | 48 | 24.33 | 1.97 | + | 0.012 |
| extracellular space | 1558 | 84 | 49.55 | 1.7 | + | 0.00246 |

**Table S2.** Classification of the fast-evolving genes (w>0.25) in solenodon genome using PANTHER Overrepresentation Test (release 20160715) and GO Ontology database (Released 2017-02-28) based on the *Mus musculus* genes.  Genes are only represented if P<0.05 after the Bonferroni correction for multiple testing (Treangen and Salzberg, 2012)

|  | **# genes in reference** | **Observed #** | **Expected#** | **Fold Enrichment** | **Positive/negative** | **P value** |
| --- | --- | --- | --- | --- | --- | --- |
| **GO biological process** | | | | | | |
| inflammatory response | 406 | 31 | 10.73 | 2.89 | + | 0.002 |
| response to stress | 2771 | 118 | 73.24 | 1.61 | + | 0.000998 |
| positive regulation of secretion | 417 | 29 | 11.02 | 2.63 | + | 0.0296 |
| DNA metabolic process | 620 | 39 | 16.39 | 2.38 | + | 0.00749 |
| cellular response to DNA damage stimulus | 598 | 37 | 15.81 | 2.34 | + | 0.0207 |
| cellular response to stress | 1244 | 63 | 32.88 | 1.92 | + | 0.00693 |
| **GO molecular function** | | | | | | |
| neuropeptide hormone activity | 27 | 7 | 0.71 | 9.81 | + | 0.025 |
| cytokine receptor activity | 88 | 15 | 2.33 | 6.45 | + | 0.000062 |
| cytokine activity | 210 | 18 | 5.55 | 3.24 | + | 0.048 |
| enzyme inhibitor activity | 385 | 26 | 10.18 | 2.56 | + | 0.0489 |
| **GO cellular component** | | | | | | |
| cell surface | 927 | 47 | 24.5 | 1.92 | + | 0.0277 |
| membrane-bounded organelle | 11251 | 366 | 297.38 | 1.23 | + | 0.0000108 |
| cytoplasm | 10108 | 327 | 267.17 | 1.22 | + | 0.000622 |
| intracellular organelle | 11117 | 347 | 293.84 | 1.18 | + | 0.00869 |

**Table S3.** Microsatellite alleles discovered in genomes of two solenodon *subspecies Solenodon paradoxus paradoxus* (northern) and *S. p. woodi* (southern).

|  | | **Assembly A** | | **Assembly B** | | **Assembly C** |
| --- | --- | --- | --- | --- | --- | --- |
| **ALL MICROSATELLITE LOCI DISCOVERED** | | | | | | |
| Total number of loci detected and computationally varified in all samples | | 8263 | | 9153 | | 8897 |
| Total number of loci verified with no variation | | 7666 | | 8514 | | 8315 |
| Loci fixed in *S. p. wodii* | | 7857 | | 8727 | | 8519 |
| Loci fixed in *S. p. paradoxus* | | 7931 | | 8803 | | 8584 |
| **Total number of loci variable in *S. p. wodii*** | | 2471 | | 2660 | | 2362 |
| **VARIABLE ALLELES** | | | | | | |
| Genotyped in all with variation in *S. p. wodii* | | 406 | | 426 | | 378 |
| Genotyped in all with variation in *S. p. paradoxus* | | 332 | | 350 | | 313 |
| Overlap - genotyped in all with variation in both | | 180 | | 182 | | 153 |
| **Total number of loci with at least one alternative variant in genotypes in all samples** | | **597** | | **639** | | **582** |
| **UNIQUE ALLELES IN EACH SUBSPECIES** | | | | | | |
| *S. p. paradoxus* contains unique alt allele, not present in *S. p. wodii* | 295 | | 323 | | 295 | |
| *S. p. wodii* contains unique alt allele, not present in *S. p. paradoxus* | 399 | | 429 | | 389 | |
| **FIXED ALLELES** | | | | | | |
| Fixed loci in *S. p. wodii* with variation in *S. p. paradoxus* | | 152 | | 168 | | 160 |
| Fixed loci in *S. p. paradoxus* with variation in *S. p. wodii* | | 226 | | 244 | | 225 |
| **Loci that fixed on different allele in each of the subspecies** | | **39** | | **45** | | **44** |

**Table S4.** Locations for the samples used in this study as described in **Figure 2**.

Adopted from Brandt et al., (2016)

| **Subspecies** | **Province** | **Locality** | **Longitude** | **Lattitude** | **Sex** | **Weight (g)** | **Sample Name** |
| --- | --- | --- | --- | --- | --- | --- | --- |
| *S.p.woodi* | Pedernales | La Cañada del Verraco | N 18^o^ 09’ 9.64” | W 710 43’ 12.0” | M | 579 | Wild K |
| *S.p. woodi* | Pedernales | La Cañada del Verraco | N 18^o^ 09’ 9.64” | W 710 43’ 12.0” | M | 1020 | Wild L |
| *S.p. woodi* | Pedernales | El Manguito -1 | N 18^0^ 06’ 36.6” | W 710 43’ 3.58” | M | 1270 | Wild M |
| *S.p. woodi* | Pedernales | El Manguito -1 | N 18^0^ 06’ 36.6” | W 710 43’ 3.58” | F | 1420 | Wild N |
| *S.p. woodi* | Pedernales | El Manguito - 2 | N 18^0^ 07’ 6.5” | W 710 43’ 14.7” | F | 1120 | Wild O |
| *S.p.paradoxus* | Espaillat | Cordillera Septentrional | Zoo |  | M |  |  |

**Table S5.** Sequencing data for the samples used in this study

| **Subspecies** | **Locality** | **Sample Name** | **Total**  **Bases** | **Read**  **Count** | **GC**  **(%)** | **AT**  **(%)** | **Q20**  **(%)** | **Q30**  **(%)** |
| --- | --- | --- | --- | --- | --- | --- | --- | --- |
| *S.p.woodi* | La Cañada del Verraco | Wild K | 13,305,687,076 | 131,739,476 | 39.99 | 60.01 | 97.18 | 92.88 |
| *S.p. woodi* | La Cañada del Verraco | Wild L | 14,443,700,536 | 143,006,936 | 39.83 | 60.17 | 97.3 | 93.16 |
| *S.p. woodi* | El Manguito -1 | Wild M | 15,350,869,204 | 151,988,804 | 39.4 | 60.6 | 97.51 | 92.79 |
| *S.p. woodi* | El Manguito -1 | Wild N | 15,539,349,950 | 153,854,950 | 39.23 | 60.77 | 97.62 | 93.04 |
| *S.p. woodi* | El Manguito - 2 | Wild O | 14,672,605,926 | 145,273,326 | 39.45 | 60.55 | 96.87 | 92.08 |
| *S.p.paradoxus* | Cordillera Septentrional | Zoo I | 7,016,027,208 | 52,358,412 |  |  |  |  |
