# Supplementary material for: Innovative assembly strategy contributes to understanding the evolution and conservation genetics of the endangered Solenodon paradoxus from the island of Hispaniola
Source: Gigascience. 2018 Mar 16;7(6):giy025. doi: 10.1093/gigascience/giy025 (PMC6009670; doi:10.1093/gigascience/giy025)
Supplement: Response_to_Reviewer_Comments_Original_Submission.pdf [file giy025_response_to_reviewer_comments_original_submission.pdf]

Author's Response To Reviewer Comments

REVIEWER #1

Grigorev et al demonstrate how low coverage sequencing of several individuals with low levels of heterozygosity, combined with a string-graph based assembly approach, can produce a useful, quality, genome assembly. The different assembly approaches used were thoroughly tested before drawing the conclusion that a string-graph based approach produced a higher quality assembly. The authors were sensitive to the limitations of this approach more generally, noting its being computationally intensive makes it generally a more undesirable approach for higher sequence coverage genomes as is its sensitivity to sequence variation. Nevertheless, a strong case was made for using this approach for low coverage genomes, particularly for threatened species were obtaining material for high coverage sequencing may be an issue.
The utility of the data presented in this manuscript was explored in several ways, including assisting in determining the phylogenetic position, identification of potential venom genes and microsatellites for conservation genetics. Although the analysis of the venom genes is challenged by the lack of a saliva sample for transcriptomics, the comparative genomic analysis provides a good starting point for future studies if such a sample should become available in the future.
Overall, I felt that the authors presented an excellent approach for utilizing low genome coverage data while recognizing the limitations of their data (e.g. the limitations of using a low coverage genome to accurately assess repeat content of the genome) and therefore, the discussion of their findings was appropriate and not overstated.

Reviewer Comment 1.1: Prior to publication, the authors should check for typos in Tables S3 and S4 (e.g. varified should be verified; wodii should be woodi etc).
Action: We have asked a professional copy editor to run through the last version of the paper and have corrected the typos in the tables and elsewhere.

REVIEWER #2

This manuscript reports the whole genome sequencing of the Hispaniolan solenodon (Solenodon paradoxus), an emblematic mammalian taxa of great conservation value. The genome sequence has been obtained by mixing 5 individuals from the southern subspecies (S. p. woodi) to reach a mean coverage of about 26x. For comparative purpose, the authors also obtained shallow genome sequencing (5x) for one individual of the Northern species (S. p. paradoxus). These first genomic data are particularly valuable because this species represents an isolated branch of the mammalian tree that diverged early from other eulipothyphan insectivores and is at conservation risk. The genomic data reported in this manuscript will therefore provide an important resource for the conservation of this endangered species. Also, given the relatively low coverage obtained even when mixing individuals, the authors explored different strategies of genome assembly and compared a classical de Brujn graph assembler (SOAPdenovo) with a string-graph based assembler strategy (Fermi), which in this case provided a better assembly both in terms of genome structure and gene annotation. These observations will be useful for assembling other genomes for which only low coverage sequencing data are currently available.
The manuscript is densely written and it would need some editing to improve some particularly

long sentences (e.g. page 4 lines 56-63; page 15; lines 2-12) and for correcting a number of remaining typos in both the main text (e.g. page 10, lines 31-33) and in figure legends. Moreover, I have some major comments and suggestions for improvement on some evolutionary analyses.

Action: We have asked a professional copy editor to run through the last version of the paper. We hope that at this point the vast majority of the types are eliminated.

Reviewer Comment 2.1. First, I noticed some tree-thinking errors in referring to the phylogenetic position and distinctiveness of Solenodon in placental mammals.
In the abstract it is stated that: "The genus occupies one of the most ancient branches among the placental mammals". As a living species, the solenodon does not occupy an ancient branch of the placental mammal tree. I would rather say: "The genus represents an isolated branch in the tree of placental mammals, which diverged early from other lipothyplan insectivores".

Page 3 lines 10-12: "Phenotypically, solenodons resemble shrews (Figure 1), but molecular evidence indicates that they are basal to all other eulipotyphlan insectivores, having split from other placental mammals in the Cretaceous Period". As currently written, this sentence suggests that eulipotyphlan insectivores are the sister group to all other placentals. Also, I would avoid referring to the term "basal" by rather writing: "Phenotypically, solenodons resemble shrews (Figure 1), but molecular evidence indicates that they are actually the sister-group of all other eulipotyphlan insectivores from which they split in the Cretaceous Period".
Page 15 lines 4-6: Same idea here, solenodons are not "one of the earliest branches that split from the placental mammal tree".

Action: All the indicated changes have been made in the text. A separate, PDF with tracked changes has been generated and communicated to the Editor.

Reviewer Comment 2.2. I think that the rationale for mixing individuals should be made clear from the beginning. Indeed, in the current version, homozygosity and low genetic diversity are a priori assumed by the authors to be hallmarks of island populations and endangered and/or endemic species. However, we are indeed far from being able to a priori predict genetic diversity of a species given our currently relatively limited understanding of its determinants. In particular, no clear correlation has yet been found between genetic diversity and conservation status and/or population size (see Ellegren & Galtier 2016 Nat. Rev. Genet.). I thus would like to see a proper demonstration that it's actually the case for the S. paradoxus woodi. As it has been done previously (Brandt et al. 2017), comparing the mitochondrial genomes of the different individuals could be used to evaluate genetic diversity. It might thus be good to put more emphasis on the results of this previous paper based on the sequencing of the same individuals in order to justify the choice of mixing individuals in the present study (e.g. page 3 lines 57-62). As currently presented, the choice of mixing 5 individuals sequenced at low coverage (5x) for assembling a composite reference genome appears awkward. I hardly understand why such a rational has been chosen instead of sequencing a reference individual at deeper coverage? Is it a problem of biological material availability/quality?

Actions: This is a valid point, as it clarifies the reasons why the assembly was done this way.

There are two reasons, first was the sample availability, second was the low homozygosity. We have made the following changes to introduce these clarifications (in the main text, you can see the modifications highlighted in yellow):

Page 4. Added a paragraph:
"Morphometric studies suggest that southern and northern Hispaniolan solenodons may be distinctive enough to be considered separate subspecies [2,13,14], a notion supported by recent mitochondrial DNA studies [11,15]. It is important to note that although population differences between the northern and southern populations was fixed, there were only a few mutational steps that differentiated the two subspecies [11]. Moreover, the southern Hispaniolan solenodons appear to have less genetic diversity than those in the north, so much so, that the control region sequences of tall five southern specimens (the same individuals used in this study) were identical [11]. This may indicate that Hispaniolan solenodons have one of the lowest known levels of genetic diversity among mammals.."

Page 5. Modified a sentence to strengthen the justification:
"The predicted mean genome coverage was approximately 5x for each sample (Figure 3), which is too low for individual de-novo genome assembly. However, because of the extremely low levels of genetic diversity suggested by the earlier study of the mitochondrial DNA in the southern subspecies [11], and in order to increase the average depth of coverage, the reads from the five samples were combined into a single data set. "

Reviewer Comment 2.3. The assembly obtained is said to be comparable to other available mammalian assemblies but only 4,416 single-copy orthologous genes have been identified in solenodon whereas 9,416 such genes can be found in Sorex and 10,773 in Erinaceus in the latest version of the OrthoMaM database. These figures also seem to be contradictory with the assertion that "the assembly provided annotation for more than 95% of the genes" (page 16 lines 56-57). Please clarify.

Comment: Single copy orthologs are used so that recent paralogs, duplications or copy number polymorphisms do not confuse evolutionary comparisons before better information can be obtained about the genes and their sequence evolution in this species.

Action: To clarify this point, we modified a sentence on page 16 to reflect the concerns of the reviewer.
"More importantly, the assembly provided complete or partial annotation for more than 95% of the genes based on the evolutionarily-informed expectations of gene content from near-universal single-copy orthologs selected from OrthoDB v9 by BUSCO [26]. Among these, 4416 single copy genes that have clear one-to-one orthologs between species (single copy orthologs)[91][92] were selected for the subsequent comparative analysis between genes in different mammalian species."

Reviewer Comment 2.4. I don't really understand the justification of using only 4-fold degenerate sites to estimate divergence dates. These positions are indeed expected to be neutral, and maybe more clocklike, but they are also potentially highly saturated because of the accumulation of multiple synonymous substitutions. Substitutional saturation at third codon

positions might result in biased divergence time estimates because of substitution rate underestimation. Therefore, I would actually suggest estimating divergence times on this dataset after excluding the 3rd codon positions or the 4-fold degenerate sites to limit the impact of substitutional saturation. I would also like to see the ML phylogram inferred from the amino acid dataset being presented as a first panel of Figure 5 with branch length estimates in order to illustrate evolutionary rate heterogeneity among lineages. In this context, it would also be important to indicate which model of rate variation (or clock relaxation) has been used in the MCMCtree dating analyses. I would finally be nice to discuss in this paragraph the potential causes behind the discrepancies observed between these divergence estimates and the younger ones obtained by Sato et al. (2016).

Comment: Thank you for pointing out the missing ML tree. It was described in the manuscript but never shown. As for the divergence time estimation was made by the MCMCtree tool from the software package PAML [46] with the HKY+G model of nucleotide substitutions and 2,200,000 generations of MCMC (of which the first 200,000 generations were discarded as burn-in). We now added the tree and described this in the legend to Figure 5. The tree was built using homologous gene sequences that were found in all species, and most estimates of the neutral rate of change from protein-coding sequences focus only on four-fold degenerate sites. Yes, the substitutions in third codon positions have a big impact across great evolutionary distances for example, between different lineages of vertebrates (e.g. birds vs. mammals), and usage of these sites to estimate divergence dates could potentially introduce bias into our results. However, within the mammalian clade the distances are small enough that this saturation is usually ignored because the selection on first and second codon positions have much stronger effect. This same justification was recently used for divergence time estimates in other mammalian genome paper, specifically the cheetah (Dobrynin et al., 2015) and the pangolin (Choo et al, 2016), to give a few examples.

Choo, S. W., Rayko, M., Tan, T. K., Hari, R., Komissarov, A., Wee, W. Y., ... & Wilson, R. K. (2016). Pangolin genomes and the evolution of mammalian scales and immunity. Genome research, 26(10), 1312-1322.

Dobrynin R., et al. 2015. Genomic legacy of the African cheetah, Acinonyx jubatus. Genome Biology 16:277

Action: We have added the ML phylogeny tree to the Figure 5 which now contains panels A and B. The new legend reads:

"Figure 5. Phylogenetics of Solenodon paradoxus from whole-genome data. A. Maximum likelihood phylogeny showing branch length. The tree was built using RAxML [45] with the PROTGAMMAAUTO option and the JTT fitting model tested with 1,000 bootstrap replications. B. Divergence time estimates based on 461,539 four-fold degenerate sites from the codon alignments of single-copy orthologs and on fossil-based priors (Table 5). The divergence time estimation was made by the MCMCtree tool from the software package PAML [46] with the HKY+G model of nucleotide substitutions and 2,200,000 generations of MCMC (of which the first 200,000 generations were discarded as burn-in). The 95% confidence intervals are given in square brackets and depicted as semitransparent boxes around the nodes. The inferred divergence time of S. paradoxus from other mammals is 73.6 Mya (95% confidence interval of 61.4-88.2 Mya). "

As to the issue with saturation of the sites, just to make sure that this was not the case in our unusual lineage, a test of substitution saturation (Xia et al. 2003; Xia and Lemey 2009) was performed using DAMBE6 (Xia X. 2017) for both all 3rd codon positions and only 4-fold degenerated sites. In both cases the Iss (index of substitution saturation) was significantly lower than threshold value for both symmetrical and asymmetrical trees (Table N) indicating low saturation level. Therefore, saturation was not detected for any of the 3d positions nor for the 4-fold degenerated sites.

Type Iss Iss.c (symmetrical tree) Iss.c (assymetrical tree)
all 3rd codon positions 0.3565 0.8472 0.7402
4-fold degenerated sites 0.4206 0.8467 0.7402

Xia, X., Z. Xie, M. Salemi, L. Chen, Y. Wang. 2003. An index of substitution saturation and its application. Molecular Phylogenetics and Evolution 26:1-7.
Xia, X. and Lemey, P. 2009. Assessing substitution saturation with DAMBE. Pp. 615-630 in Philippe Lemey, Marco Salemi and Anne-Mieke Vandamme, eds. The Phylogenetic Handbook: A Practical Approach to DNA and Protein Phylogeny. 2nd edition Cambridge University Press.
Xia X. 2017. DAMBE6: New tools for microbial genomics, phylogenetics and molecular evolution. Journal of Heredity 108(4):431-437.

Action: We added the following text (Page 10):
"A test for substitution saturation (Xia et al. 2003; Xia and Lemey 2009) was performed using DAMBE6 (Xia X. 2017) for both all 3rd codon positions and only 4-fold degenerated sites. In both cases the Iss (index of substitution saturation) was significantly lower than threshold value for both symmetrical and asymmetrical trees (Table N) indicating low saturation level. Therefore, saturation was not detected for any of the 3d positions nor for the 4-fold degenerated sites."
As to the issue for discrepancies in the divergence time estimates: some of our authors (Roca) have address this question specifically in a manuscript that is now accepted (Springer et al., 2018).. We added text on page 10 to refer to this new publication:
"According to this analysis, S. paradoxus diverged from other mammals 73.6 Mya (95% confidence interval of 61.4-88.2 Mya). This is in accordance with earlier estimates based on nuclear and mitochondrial sequences (e.g., Roca et al., 2004; Brandt et al., 2017) as reviewed by Springer et al. (Springer et al., 2018). This date is also much older than the timeframe of molecular estimates of divergence times between most island taxa and their closest mainland relatives [52]."

Reviewer Comment 2.5 As far I understand from the information provided on analyses performed using codeml, the dN/dS ratio of each of the 4,416 single-copy orthologous genes has been inferred globally from the codon alignments including the 11 species presented in Figure 5. If this is correct, I don't really see the rationale for performing such an analysis that is entirely dependent of the arbitrary choice of species that were included in the dataset. Identifying genes that are evolving under positive selection globally is of limited interest in the context of this manuscript focused on the evolution of solenodon. I would rather suggest estimating dN/dS by gene focusing on the branch leading to Solenodon using the branch model in codeml. This would allow pinpointing genes that have been positively selected during the evolution of solenodons.

Action: We have rerun the dN/dS comparison analysis using only the four species of eulypotiphlan insectivores Figure 5. The smallest comparison that can be used in this analysis is 4 species. In the 4 - species comparison, we used the following species: Erinaceus europaeus, Sorex araneus. Condylura cristata, and S. paradoxus. Unfortunately, in reality, this results in increased numbers of outliers, making it very difficult to interpret, as it is not clear what the values of dN/dS mean (see the graph in the bottom right corner). Since shifting to 4 species introduces a large number of big values of dN/dS, we would like to keep the 10 species comparison, even though it may not be as insightful.

Additional comments:
Page 5 line 9: Explain what is the "general field protocol".
Action: The protocol was described earlier in Brandt et al. 2016. Now we added the corresponding reference in that line.

Page 5 line 28: Indicate Illumina read length used for sequencing in the main manuscript.
Action: Added the following sentence (page 5): "The five S. p. woodi samples were sequenced using Hiseq2000 technology (Illumina Inc.), resulting in an average of 151,783,327paired-end reads 101 bp long, or 15.33Gb of sequence data, per individual."
