# Supplementary material for: Innovative assembly strategy contributes to understanding the evolution and conservation genetics of the endangered Solenodon paradoxus from the island of Hispaniola
Source: Gigascience. 2018 Mar 16;7(6):giy025. doi: 10.1093/gigascience/giy025 (PMC6009670; doi:10.1093/gigascience/giy025)
Supplement: Response_to_Reviewer_Comments_Revision_1.pdf [file giy025_response_to_reviewer_comments_revision_1.pdf]

Author's Response To Reviewer Comments

Dear Dr. Edmunds

Thank you very much for getting back to me so quickly. I carefully went through your comments and addressed all the issues you have raised. Here are the answers to your comments line-by-line. I hope that you will find these satisfactory, but if anything outstanding remains, please let me know as soon as possible.

Sincerely yours

Taras Oleksyk, UPR-M

Line 134
Are there also voucher numbers?

There are no voucher numbers, as the animals were released on location.

Line 151
This is a bit sparse

Information is added to M&M as requested

Line 253
Make sure this citation remains

Citations were fixed with the new addition of the GigaScience database

Line 267
Missing citation in the references

added
E. P. Nawrocki and S. R. Eddy, Infernal 1.1: 100-fold faster RNA homology searches, Bioinformatics 29:2933-2935 (2013).

Line 284
Can you make sure the scripts are in GigaDB or zenodo?

I have asked access to the Progressive Cactus scripts. They are are here:
https://zenodo.org/record/1189347#.Wp6Jt2aB3sE
can this reference be added to GIGADB?

Line 652
Citation missing in references

Added citation 12

Line 682
Are there reference numbers for these as please include them? If there are voucher numbers and details please include these too

The text was added: "All the required collection and permits had been obtained before any field work was started. The samples have been collected and exported in compliance with Export Permit No: VAPB-00909 (Dominican Republic Environment and Natural Resources Ministry Viceministry of Protected Areas and Biodiversity Department of Biodiversity) and imported in compliance with CITES/ESA Import permit number 14US84465A/9 (University of Illinois Board of Trustees)."

Line 689
What model of HiSeq? 2500? 4000? How did library construction work? Did you use PE reads and what size?

Added: "Sequences for S. p. woodi were generated by Illumina HiSeq 2000 (Illumina Inc) with 100bp paired-end reads."

Line 730
Make sure this is cited correctly with the full DOI. The curators should give you this when you check the mockup (don't use that). Please also include the accession and zenodo details i f the code is going there

Added the correct DOI to the database, also the accession numbers

ENA: NKTL01000000
PROJECT: PRJNA368679

Additional information: http://doi.org/10.5281/zenodo.1185377

I would also ask to add and addition Zenodo repository here for Progressive Cactus scripts
https://zenodo.org/record/1189347#.Wp6Jt2aB3sE
