# Supplementary material for: Innovative assembly strategy contributes to understanding the evolution and conservation genetics of the endangered Solenodon paradoxus from the island of Hispaniola
Source: Gigascience. 2018 Mar 16;7(6):giy025. doi: 10.1093/gigascience/giy025 (PMC6009670; doi:10.1093/gigascience/giy025)
Supplement: Reviewer_1_Report_(Original_Submission) -- Janine Deakin — 29 Aug 2017 Reviewed [file giy025_reviewer_1_report_(original_submission).pdf]

Reviewer Report

Title: Innovative assembly strategy contributes to understanding the evolution and conservation genetics of the endangered Solenodon paradoxus from the island of Hispaniola

Version: Original Submission **Date:** 29 Aug 2017

Reviewer name: Janine Deakin

Reviewer Comments to Author:

Grigorev et al demonstrate how low coverage sequencing of several individuals with low levels of heterozygosity, combined with a string-graph based assembly approach, can produce a useful, quality, genome assembly. The different assembly approaches used were thoroughly tested before drawing the conclusion that a string-graph based approach produced a higher quality assembly. The authors were sensitive to the limitations of this approach more generally, noting its being computationally intensive makes it generally a more undesirable approach for higher sequence coverage genomes as is its sensitivity to sequence variation. Nevertheless, a strong case was made for using this approach for low coverage genomes, particularly for threatened species were obtaining material for high coverage sequencing may be an issue.

The utility of the data presented in this manuscript was explored in several ways, including assisting in determining the phylogenetic position, identification of potential venom genes and microsatellites for conservation genetics. Although the analysis of the venom genes is challenged by the lack of a saliva sample for transcriptomics, the comparative genomic analysis provides a good starting point for future studies if such a sample should become available in the future.

Overall, I felt that the authors presented an excellent approach for utilizing low genome coverage data while recognizing the limitations of their data (e.g. the limitations of using a low coverage genome to accurately assess repeat content of the genome) and therefore, the discussion of their findings was appropriate and not overstated.

Minor comment:
Prior to publication, the authors should check for typos in Tables S3 and S4 (e.g. varified should be verified; wodii should be woodi etc).

Level of Interest

Please indicate how interesting you found the manuscript: An article of importance in its field

Quality of Written English

Please indicate the quality of language in the manuscript: Acceptable

Declaration of Competing Interests

Please complete a declaration of competing interests, considering the following questions:

- Have you in the past five years received reimbursements, fees, funding, or salary from an organisation that may in any way gain or lose financially from the publication of this manuscript, either now or in the future?
- Do you hold any stocks or shares in an organisation that may in any way gain or lose financially from the publication of this manuscript, either now or in the future?
- Do you hold or are you currently applying for any patents relating to the content of the manuscript?
- Have you received reimbursements, fees, funding, or salary from an organization that holds or has applied for patents relating to the content of the manuscript?
- Do you have any other financial competing interests?
- Do you have any non-financial competing interests in relation to this paper?

If you can answer no to all of the above, write 'I declare that I have no competing interests' below. If your reply is yes to any, please give details below.

I declare that I have no competing interests

I agree to the open peer review policy of the journal. I understand that my name will be included on my report to the authors and, if the manuscript is accepted for publication, my named report including any attachments I upload will be posted on the website along with the authors' responses. I agree for my report to be made available under an Open Access Creative Commons CC-BY license (http://creativecommons.org/licenses/by/4.0/). I understand that any comments which I do not wish to be included in my named report can be included as confidential comments to the editors, which will not be published.

I agree to the open peer review policy of the journal

To further support our reviewers, we have joined with Publons, where you can gain additional credit to further highlight your hard work (see: https://publons.com/journal/530/gigascience). On publication of this paper, your review will be automatically added to Publons, you can then choose whether or not to claim your Publons credit. I understand this statement.

Yes
