# Supplementary material for: Innovative assembly strategy contributes to understanding the evolution and conservation genetics of the endangered Solenodon paradoxus from the island of Hispaniola
Source: Gigascience. 2018 Mar 16;7(6):giy025. doi: 10.1093/gigascience/giy025 (PMC6009670; doi:10.1093/gigascience/giy025)
Supplement: Reviewer_2_Report_(Original_Submission) -- Frederic Delsuc — 18 Sep 2017 Reviewed [file giy025_reviewer_2_report_(original_submission).pdf]

Reviewer Report

Title: Innovative assembly strategy contributes to understanding the evolution and conservation genetics of the endangered Solenodon paradoxus from the island of Hispaniola

Version: Original Submission **Date:** 18 Sep 2017

Reviewer name: Frederic Delsuc

Reviewer Comments to Author:

This manuscript reports the whole genome sequencing of the Hispaniolan solenodon (Solenodon paradoxus), an emblematic mammalian taxa of great conservation value. The genome sequence has been obtained by mixing 5 individuals from the southern subspecies (S. p. woodi) to reach a mean coverage of about 26x. For comparative purpose, the authors also obtained shallow genome sequencing (5x) for one individual of the Northern species (S. p. paradoxus). These first genomic data are particularly valuable because this species represents an isolated branch of the mammalian tree that diverged early from other eulipothyphan insectivores and is at conservation risk. The genomic data reported in this manuscript will therefore provide an important resource for the conservation of this endangered species. Also, given the relatively low coverage obtained even when mixing individuals, the authors explored different strategies of genome assembly and compared a classical de Brujn graph assembler (SOAPdenovo) with a string-graph based assembler strategy (Fermi), which in this case provided a better assembly both in terms of genome structure and gene annotation. These observations will be useful for assembling other genomes for which only low coverage sequencing data are currently available.

The manuscript is densely written and it would need some editing to improve some particularly long sentences (e.g. page 4 lines 56-63; page 15; lines 2-12) and for correcting a number of remaining typos in both the main text (e.g. page 10, lines 31-33) and in figure legends. Moreover, I have some major comments and suggestions for improvement on some evolutionary analyses.

1. First, I noticed some tree-thinking errors in referring to the phylogenetic position and distinctiveness of Solenodon in placental mammals.
In the abstract it is stated that: "The genus occupies one of the most ancient branches among the placental mammals". As a living species, the solenodon does not occupy an ancient branch of the placental mammal tree. I would rather say: "The genus represents an isolated branch in the tree of placental mammals, which diverged early from other lipothyplan insectivores".
Page 3 lines 10-12: "Phenotypically, solenodons resemble shrews (Figure 1), but molecular evidence indicates that they are basal to all other eulipotyphlan insectivores, having split from other placental mammals in the Cretaceous Period". As currently written, this sentence suggests that eulipotyphlan insectivores are the sister group to all other placentals. Also, I would avoid referring to the term "basal" by rather writing: "Phenotypically, solenodons resemble shrews (Figure 1), but molecular evidence indicates that they are actually the sister-group of all other eulipotyphlan insectivores from which they

split in the Cretaceous Period".
Page 15 lines 4-6: Same idea here, solenodons are not "one of the earliest branches that split from the placental mammal tree".

2. I think that the rationale for mixing individuals should be made clear from the beginning. Indeed, in the current version, homozygosity and low genetic diversity are a priori assumed by the authors to be hallmarks of island populations and endangered and/or endemic species. However, we are indeed far from being able to a priori predict genetic diversity of a species given our currently relatively limited understanding of its determinants. In particular, no clear correlation has yet been found between genetic diversity and conservation status and/or population size (see Ellegren & Galtier 2016 Nat. Rev. Genet.). I thus would like to see a proper demonstration that it's actually the case for the S. paradoxus woodi. As it has been done previously (Brandt et al. 2017), comparing the mitochondrial genomes of the different individuals could be used to evaluate genetic diversity. It might thus be good to put more emphasis on the results of this previous paper based on the sequencing of the same individuals in order to justify the choice of mixing individuals in the present study (e.g. page 3 lines 57-62). As currently presented, the choice of mixing 5 individuals sequenced at low coverage (5x) for assembling a composite reference genome appears awkward. I hardly understand why such a rational has been chosen instead of sequencing a reference individual at deeper coverage? Is it a problem of biological material availability/quality?

3. The assembly obtained is said to be comparable to other available mammalian assemblies but only 4,416 single-copy orthologous genes have been identified in solenodon whereas 9,416 such genes can be found in Sorex and 10,773 in Erinaceus in the latest version of the OrthoMaM database (http://www.orthomam.univ-montp2.fr/orthomam/html/index.php). These figures also seem to be contradictory with the assertion that "the assembly provided annotation for more than 95% of the genes" (page16 lines 56-57). Please clarify.

4. I don't really understand the justification of using only 4-fold degenerate sites to estimate divergence dates. These positions are indeed expected to be neutral, and maybe more clocklike, but they are also potentially highly saturated because of the accumulation of multiple synonymous substitutions. Substitutional saturation at third codon positions might result in biased divergence time estimates because of substitution rate underestimation. Therefore, I would actually suggest estimating divergence times on this dataset after excluding the 3rd codon positions or the 4-fold degenerate sites to limit the impact of substitutional saturation. I would also like to see the ML phylogram inferred from the amino acid dataset being presented as a first panel of Figure 5 with branch length estimates in order to illustrate evolutionary rate heterogeneity among lineages. In this context, it would also be important to indicate which model of rate variation (or clock relaxation) has been used in the MCMCtree dating analyses. I would finally be nice to discuss in this paragraph the potential causes behind the discrepancies observed between these divergence estimates and the younger ones obtained by Sato et al. (2016).

5. As far I understand from the information provided on analyses performed using codeml, the dN/dS

ratio of each of the 4,416 single-copy orthologous genes has been inferred globally from the codon alignments including the 11 species presented in Figure 5. If this is correct, I don't really see the rationale for performing such an analysis that is entirely dependent of the arbitrary choice of species that were included in the dataset. Identifying genes that are evolving under positive selection globally is of limited interest in the context of this manuscript focused on the evolution of solenodon. I would rather suggest estimating dN/dS by gene focusing on the branch leading to Solenodon using the branch model in codeml. This would allow pinpointing genes that have been positively selected during the evolution of solenodons.

Additional comments:
Page 5 line 9: Explain what is the "general field protocol".
Page 5 line 28: Indicate Illumina read length used for sequencing in the main manuscript.

Level of Interest

Please indicate how interesting you found the manuscript: An article of importance in its field

Quality of Written English

Please indicate the quality of language in the manuscript: Needs some language corrections before being published

Declaration of Competing Interests

Please complete a declaration of competing interests, considering the following questions:

- Have you in the past five years received reimbursements, fees, funding, or salary from an organisation that may in any way gain or lose financially from the publication of this manuscript, either now or in the future?
- Do you hold any stocks or shares in an organisation that may in any way gain or lose financially from the publication of this manuscript, either now or in the future?
- Do you hold or are you currently applying for any patents relating to the content of the manuscript?
- Have you received reimbursements, fees, funding, or salary from an organization that holds or has applied for patents relating to the content of the manuscript?
- Do you have any other financial competing interests?
- Do you have any non-financial competing interests in relation to this paper?

If you can answer no to all of the above, write 'I declare that I have no competing interests' below. If your reply is yes to any, please give details below.

I declare that I have no competing interests

I agree to the open peer review policy of the journal. I understand that my name will be included on my report to the authors and, if the manuscript is accepted for publication, my named report including any attachments I upload will be posted on the website along with the authors' responses. I agree for my report to be made available under an Open Access Creative Commons CC-BY license (http://creativecommons.org/licenses/by/4.0/). I understand that any comments which I do not wish to be included in my named report can be included as confidential comments to the editors, which will not be published.

I agree to the open peer review policy of the journal

To further support our reviewers, we have joined with Publons, where you can gain additional credit to further highlight your hard work (see: https://publons.com/journal/530/gigascience). On publication of this paper, your review will be automatically added to Publons, you can then choose whether or not to claim your Publons credit. I understand this statement.

Yes
